# A cross-sectional survey of farmer reported prevalence and farm management practices associated with neonatal infectious arthritis (“joint ill”) in lambs, on UK sheep farms

**DOI:** 10.3389/fvets.2024.1489751

**Published:** 2024-12-23

**Authors:** L. P. Jackson, H. M. Higgins, J. S. Duncan

**Affiliations:** Department of Livestock and One Health, Institute of Infection, Veterinary and Ecologica Science, University of Liverpool, Neston, United Kingdom

**Keywords:** lamb, neonatal infectious arthritis, joint ill, epidemiology, prevention, treatment, antibiotic

## Abstract

**Introduction:**

Neonatal infectious arthritis (NIA) is a bacterial disease of lambs in the first month of life. NIA is associated with poor animal welfare, economic losses, and prophylactic antibiotic use. Farmers report problems with NIA despite following current guidance on prevention. The aim of this study was to estimate NIA UK incidence, describe current management practices for NIA control, and farm management risk factors associated with NIA.

**Methods:**

A cross-sectional, online questionnaire of UK sheep farmers was carried out between June and October 2020. Descriptive statistics, and univariable and multivariable risk factor analysis was undertaken.

**Results:**

Of the 322 respondents, 64% reported joint ill cases in the 2020 lambing period. The median within flock incidence was 1.4% (IQR 0.8–2.6%; 95% CI, 1.2–1.6). Seventeen percent of farmers estimated their current treatment efficacy for NIA was less than 50%. Eleven percent of farmers used prophylactic or metaphylactic antibiotics in all lambs to prevent NIA occurrence. Across all flocks, risk factor associated with NIA occurrence was the number of ewes lambed (301–600; OR, 3.9; 95% CI 1.9–8.0. >600; OR, 13.7; 95% CI, 5.4–34.4). In outdoor lambing flocks, increased risk of joint ill was associated with the number of ewes lambed (>600 ewes; OR, 34.7; 95% CI, 6.6–182.7), not providing outdoor shelter to lambing ewes (OR, 3.0; 95% CI, 1.2–7.8), and not cleaning ear tags (OR, 5.7; 95% CI, 1.5–21.4). Using antibiotics as a preventative measure was associated with a reduced risk of joint ill (OR, 0.1; 95% CI, 0.01–0.4). In indoor lambing flocks, increased risk of joint ill was associated with upland flocks (OR, 3.0; 95% CI, 1.3–6.8), number of lambs born alive (501–1,000; OR, 4.1; 95% CI, 1.6–10.7. >1,000; OR, 14.0; 95% CI, 4.0–48.9), and not washing hands (OR, 3.6; 95% CI, 1.2–10.6).

**Conclusion:**

NIA was reported in indoor and outdoor lambing flocks. A pattern of risk factors associated with increasing intensity of lambing was observed. Prophylactic antibiotic use was associated with a reduced risk of NIA in outdoor flocks, however, NIA still occurred in flocks where this was practiced. More veterinary involvement is advised in the diagnosis, treatment protocols, and prevention of NIA.

## Introduction

Neonatal infectious arthritis (NIA), commonly referred to as ‘joint ill,’ is a bacterial disease affecting the joints of lambs. The most common agent responsible, *Streptococcus dysgalactiae* subspecies *dysgalactiae* (SDSD), affects lambs within the first month of life, most commonly within the first 2 weeks (1–3). The reservoirs for infection are currently incompletely understood, however, the ewe and the lambing environment are commonly hypothesised potential sources (4–6). Similarly, the route of transmission has not been determined, with wounds created during the neonatal period, such as those from tail docking and castration, and the navel suggested as bacterial entry points for dissemination of SDSD through the bloodstream (7). The clinical signs of NIA vary depending on which joints are affected, however, clinical symptoms typically include joint swelling, pain, lameness, and recumbency (8). Therefore, the disease is an important animal welfare concern for sheep farmers. Treatment and prevention of NIA is also associated with widespread use of antibiotics on sheep farms at lambing time (9). Economic losses attributed to NIA have not been well studied, however, the disease has been found to impact lamb growth rates (4, 10) and lamb mortality (4, 10).

SDSD-associated NIA (SDSD-NIA) is reported in many sheep producing countries including Australia (11), New Zealand (10), Spain (6), and Norway, where its prevalence has increased over recent years. Anecdotally, this is attributed to the intensification of farming practices, such as the increasing numbers of animals on farms and the length of housing periods (12). Survey data from 36 sheep flocks in England and Wales in 2015 (4) indicated that the mean within flock prevalence of NIA was 4% (range, 2–20%); while more recent survey data from Norway estimated between farm prevalence of NIA was 5.6%; with a farmer estimated within flock incidence of 5–20% (12). There is no recent UK estimate of between and within flock prevalence of SDSD-NIA. Current, evidenced-based and hypothesised SDSD-NIA risk factors have formed the basis of recommended farm control measures, with guidance focusing on farm hygiene practices (8). Recent risk factor studies in Norway, identified large flock size (OR, 1.3; 95% CI, 1.1–1.4 per 100 lambs), increasing lambing percentage (OR, 2.0; 95% CI, 1.1–3.5), and ear tag infections (OR, 2.6; 95% CI, 1.6–4.3) as risk factors for SDSD-NIA occurrence (12). While in a New Zealand study, lambs born later in the lambing period (OR, 5.6; 95% CI, 1.8–16.4) and lambs born in a birth area with greatest prevalence of NIA (OR, 7.7; 95% CI, 1.1–9.5) were at greatest risk of disease (10). Animal-based risk factors such as birth weight, litter size, dam age, and maternal mastitis had no association with SDSD-NIA development in this study (4, 10). The importance of colostrum and the role of failure of passive transfer (FPT) in the development of neonatal disease has been well documented (13–15). However, its role in the development of NIA is unclear and, anecdotally, outbreaks still occur on farms with good colostrum management (1).

As farm management practices and disease burden will vary between different sheep producing countries, the aim of this study was to provide an up-to-date estimate of NIA prevalence, describe UK farm management practices for NIA control, and explore risk factors associated with NIA on UK sheep farms.

## Methods

### Questionnaire design

The research was approved by the University of Liverpool Veterinary Research Ethics Committee (VREC 920). Questionnaire design was informed by reviewing the scientific literature on SDSD-NIA and clinical experience of the research team. A draft questionnaire was produced, piloted on farmers and vets (*n* = 6), and refined to produce a final version. Pilot data was not included in the final dataset. The questions were grouped into an introductory section which provided participant information, participant consent, and compliance with General Data Protection Regulations the Regulation (GDPR; EU 2016/679). Seven subsequent sections (A-G) were created to collect data on general farm demographics, sheep flock attributes, NIA cases, antibiotic use, farmer perception of risk factors for NIA, and farm management and animal level risk factors for NIA occurrence. The survey consisted of 78 questions, however, to encompass the variety of UK sheep management systems, not all participants were eligible to answer all questions. The questionnaire was designed to be answered by the participants via tick boxes, to enable speed of response and facilitate compliance with completion of the questionnaire. However, free text boxes enabled the participants to expand on their answers when applicable. The final survey was created using JISC online survey tool,^1^ a GDPR compliant electronic survey tool. The full survey is provided as Supplementary material 1. The survey was opened on the 17th of June 2020 at 9 am and closed on the 9th of October 2020 at 5 pm. To encourage participation an Apple iPad, Samsung Galaxy Tablet, and a Fortnum and Mason hamper were offered as prizes. For those participants wishing to enter the prize draw, a separate link was provided, allowing contact details to be entered while maintaining the anonymity of the survey. Throughout the survey, NIA was referred to by its common UK name ‘joint ill,’ which was defined as ‘a case of swollen joints in lambs less than one month old.’

### Farm recruitment

The target population, and therefore eligibility criteria, was UK sheep farmers who had or had not, experienced NIA problems in their flocks. Distribution of the survey link was through multiple media platforms, including through sheep industry organisational newsletters, social media, and email lists of the following organisations: Agricultural Horticultural Development Board, The Moredun Research Institute, National Sheep Association, National Animal Disease Information Service, Hybu Cig Cymru, Animal Plant and Health Agency, Sainsbury’s Farmer Development Group, University of Liverpool, Sheep Veterinary Society, and the KEPAK group. In addition, sheep industry personnel were asked to share the link to the survey on their professional social media accounts (Twitter and Facebook). Due to the way the survey was distributed, a random sample was not obtained, A sample size calculation to estimate the between flock prevalence of NIA, was based on a hypothesised between flock incidence prevalence of 50% and a population of 40,000 UK farms that graze livestock in 2020 (data not available on sheep-only units) (16). This resulted in an ideal sample size of 381 with 95% confidence interval and 5% precision.

### Data analysis

The data was exported from JISC (version 2, see Footnote 1) into Microsoft Office Excel (version 2019, Microsoft Corporation) and visually inspected for missing or uninterpretable answers. Surveys with missing responses and/or dropouts were included if they met initial inclusion criteria. Uninterpretable answers or answers in the wrong format were analysed and cleaned appropriately. Statistical analysis was conducted using Minitab 19 (Minitab, LLC, 2021) statistical software. All continuous variables were tested for normality prior to analysis using the Shapiro–Wilk test for normality and all were found to have a non-normal distribution. Following this, descriptive statistics were conducted accordingly for continuous data, using median and interquartile range.

Informed from current literature, knowledge and clinical experience of the research team and experts in the field, a causal diagram (Supplementary material 2) was produced and utilised to determine pre-existing and hypothetical relationships between risk factors and NIA presentation. From this, predictor variables were selected for univariable analysis. Prior to univariable analysis, categorical variables were examined via cross tabulation against the categorical outcome variable, “farmer reported presence of NIA in 2020 lambing season” with a “yes”/“no” binary response. Predicator variable categories were collapsed when applicable and/or biologically relevant to aid analysis, either due to eligibility issues, small counts in some levels, or to produce a categorical variable from a continuous one. Predicator variables were screened for collinearity: Cramer’s V-square (categorical nominal), Pearsons r (continuous), and Spearman’s rho (categorical ordinal). To resolve collinearity issues, predictor variables were either combined to produce one variable, or the most biologically relevant variable was taken forward. Two new variables were created, by combining information from related questions. These were; variable ‘% of lambs on the farm with NIA’ was calculated by dividing the number of cases per farm by the number of lambs born alive per farm in 2020; and the variable ‘Number of ewes per mothering pen’ was calculated by dividing the number of ewes lambed by the quantity of mothering pens.

### Model production

Univariable and multivariable analyses were conducted via binary logistical regression with the outcome variable “farmer reported presence of NIA in 2020 lambing season” with a “yes”/“no” binary response. Predicator variables with a *p* value equal to or less than 0.2 were taken forward for multivariable analysis. Multivariable models were constructed using an automated stepwise approach and the Logit link function, with alpha to enter and alpha to remove set at 0.05 and 0.1, respectively. Multivariable models were examined for goodness of fit via Hosmer-Lemeshow statistics.

### Sub-setting of data

Due to the wide variation in UK sheep farm management lambing practices, not all respondents were eligible to answer all questions regarding the lambing system (Section D of the questionnaire) and skip logic was used. Therefore, for univariable and multivariable analysis, the data was sub-set into data derived from (1) Indoor Lambing Flocks, (2) Outdoor Lambing Flocks, and (3) All Flocks.

#### Indoor lambing flocks

Indoor lambing flocks were defined as respondents who answered questions in the section relating to indoor lambing (Section D, questions 35-50a, Supplementary material 3), including all flocks which lambed entirely or in part indoors in lambing season 2020.

#### Outdoor lambing flocks

Outdoor lambing flocks were defined as respondents who answered questions in the section relating to outdoor lambing (Section D, questions 31–34a, Supplementary material 4), including all flocks which lambed entirely or in part outdoors in lambing season 2020.

#### All flocks

Not all respondents were eligible to answer all questions related to indoor and outdoor lambing (Section D). The all-flocks dataset includes all respondents and questions, with the exclusion of specific questions relating to indoor and outdoor lambing (Section D, 31-50a, Supplementary material 5).

## Results

### Response rate

Due to the online method of distribution, it was not possible to track the number of farmers who received the survey link and therefore, a response rate was unable to be calculated. In total, there were 328 responses to the survey. Six responses were removed for not meeting eligibility criteria (UK sheep farmer), resulting in a final 322 responses either in full or part to the survey. Following this initial eligibility question, no questions were forced answer and due to the nature of the survey, not all questions were answerable for all the participants, thus the number of responses varies per question. Throughout the results the number of responses for each question is indicated by (N), while the specific responses to a question is indicated by (n). Due to the breadth of survey, full results tables are provided in Supplementary material 6.

### Demographics

#### Farm enterprise attributes

**Figure 1:**
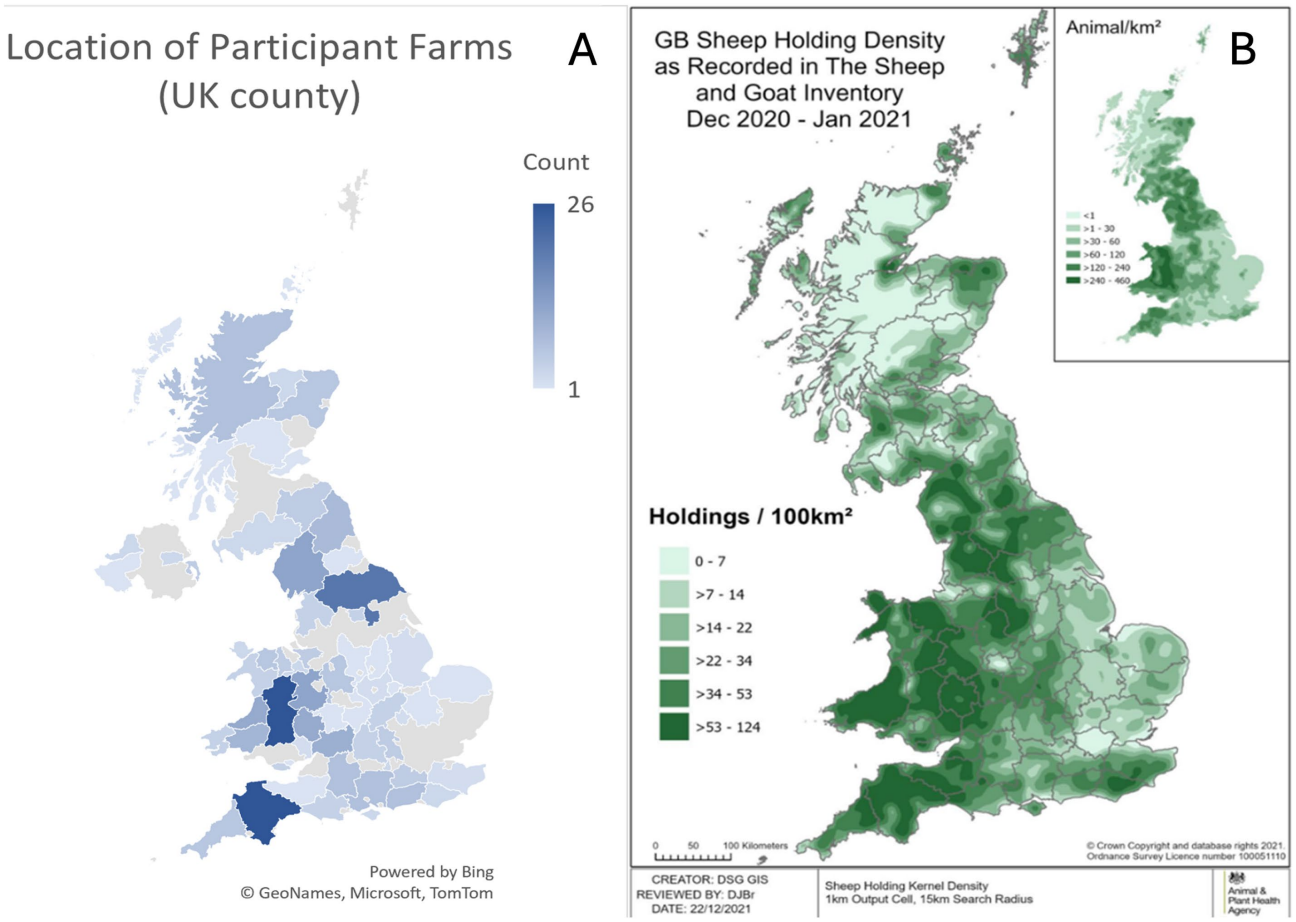
A (left) UK Geographical distribution of farms participating in the survey (*N* = 322). B (right) GB sheep holding density data for December 2020–January 2021 (26).

Section A contained questions regarding the farm enterprise. There were 322 farmers from 69 UK counties who participated in the survey, with the top three counties participating being Powys (26 farms), Devon (26 farms), and North Yorkshire (20 farms; Figure 1A). This geographic distribution of respondents is approximately representative of the GB sheep holding density (Figure 1B).

The most common type of farm participating was lowland (low lying areas of the UK where soil is arable), at 60%. This was followed by upland (areas of higher ground where the soil is less arable), at 37%, and mountain/hill, (harsh climates where soil quality is often poor) (17), at 3% (nr = 319). Of these, 11% of farms classified themselves as more than one specific category (*n* = 36). Only 5% of farmers classified themselves as organic, with 95% being non-organic. Farmers managed a median of 350 ewes (IQR, 115–4,500; nr = 321), across a median of 75 hectares of land (IQR, 28–190; nr = 315).

#### Flock attributes

As farmers often own and/or manage multiple flocks of ewes, the farmer was asked to relate all remaining questions to their main and/or largest flock. The data is summarised in Supplementary material 6, Table A.

The most common type of flock was lowland at 61%, followed by upland at 35% and mountain/hill at 4% (*N* = 316). Of the 316 responses, 10.4% of farmers classified their flock as a combination of these three categories. Farms were split into 95% non-organic to 5% organic farms.

The median flock size was 320 breeding ewes (IQR, 100–650 N = 318). Sixty one percent of farmers classified their main flock as crossbred and 39% were purebred (*N* = 317). In total, 150 different pure and cross breed combinations were described as belonging to participant flocks (*N* = 315). The five most common breeds/crosses were Texel (9%), Lleyn (7%), Mule (6%), North of England Mule (4%), and Suffolk (4%). Most farmers (96%) described the age of ewes in their flocks as mixed, with 2% answering 1–2 years old, and 2% answering ewes were over 5 years old (*N* = 319). Indoor only lambing was most common (51%), followed by lambing outdoors only (25%) and mixed lambing (24%; combination of indoors and outdoors; *N* = 319).

The most common month for lambing was April (39%), followed by March (35%). However, lambing was reported in nine different months (Nov-June, Sept). The lambing season lasted a median of 5 weeks (IQR, 1–6; *N* = 317). The median scanning percentage, defined as the total number of lamb foetuses detected by ultrasound scanning during pregnancy per breeding ewe in the flock (18), was178% (IQR, 160–190; *N* = 245). A median of 300 ewes lambed across participants farms in 2020 lambing season (IQR, 95–600; *N* = 307); with a median of 500 lambs born alive (IQR, 154–980; *N* = 304).

### Neonatal infectious arthritis

#### Farm prevalence of NIA

Of the 322 eligible respondents, 206 experienced NIA in lambing season 2020, resulting in a between farm, farmer reported prevalence of 64%. Farmers reported a median of 8.5 cases per farm (IQR, 3–20; *N* = 304). Proportionally, this equates to a median of 1.4% (IQR, 0.8–2.6%; 95% CI, 1.2–1.6) of lambs born going on to develop NIA from participant farms in 2020. The data is summarised in Supplementary material 6, Table B.

#### Characteristics of NIA cases on affected farms

Of the NIA affected farms, 63% were from flocks lambing indoors only (*N* = 204). Farmers reported that later in the lambing period as the most common time for NIA development (36%; *N* = 204) and NIA was seen most frequently in lambs aged 8–14 days old (38%; *N* = 205). Farmers described no specific type of lamb (singles, twins, triplets, orphans) as the most affected by NIA (48%; *N* = 205; Table 1). The data is summarised in Supplementary material 6, Table B.

**Table 1:** Farmer reported characteristics of the NIA cases in 2020.

Characteristic	Response
Between farm reported prevalence of NIA in 2020 (*N* = 322)	64% (206/322)
Median cases of NIA per farm in 2020 (*N* = 304)	8.5 (IQR, 3–20)
Lambs born going on to develop NIA (within flock incidence) (*N* = 304)	1.4% (IQR, 0.8–2.6; 95% CI, 1.2–1.6)
Farm systems reporting NIA (*N* = 204)	
Indoor lambing systems	63% (128/204)
Outdoor lambing systems	19% (39/204)
Mixed lambing systems	18% (37/204)
Period of lambing of NIA development (*N* = 204)	
Earlier in lambing period	12% (24/204)
Middle of lambing period	25% (51/204)
Later into lambing period	36% (73/204)
No specific time during lambing period	28% (56/204)
Age of lambs developing NIA (*N* = 205)	
0–3 days old	6% (12/205)
4–7 days old	26% (54/205)
8–14 days old	38% (78/205)
15–28 days old	24% (49/205)
Over 1 month old	6% (12/205)
Type of lambs developing NIA (*N* = 205)	
Single lambs	4% (8/205)
Twin lambs	24% (47/205)
Triplet lambs	16% (33/205)
Orphan lambs	9% (18/205)
Other type of lamb described by farmers	<1% (1/205)

#### Farmer reported causes of NIA and treatment

**Figure 2:**
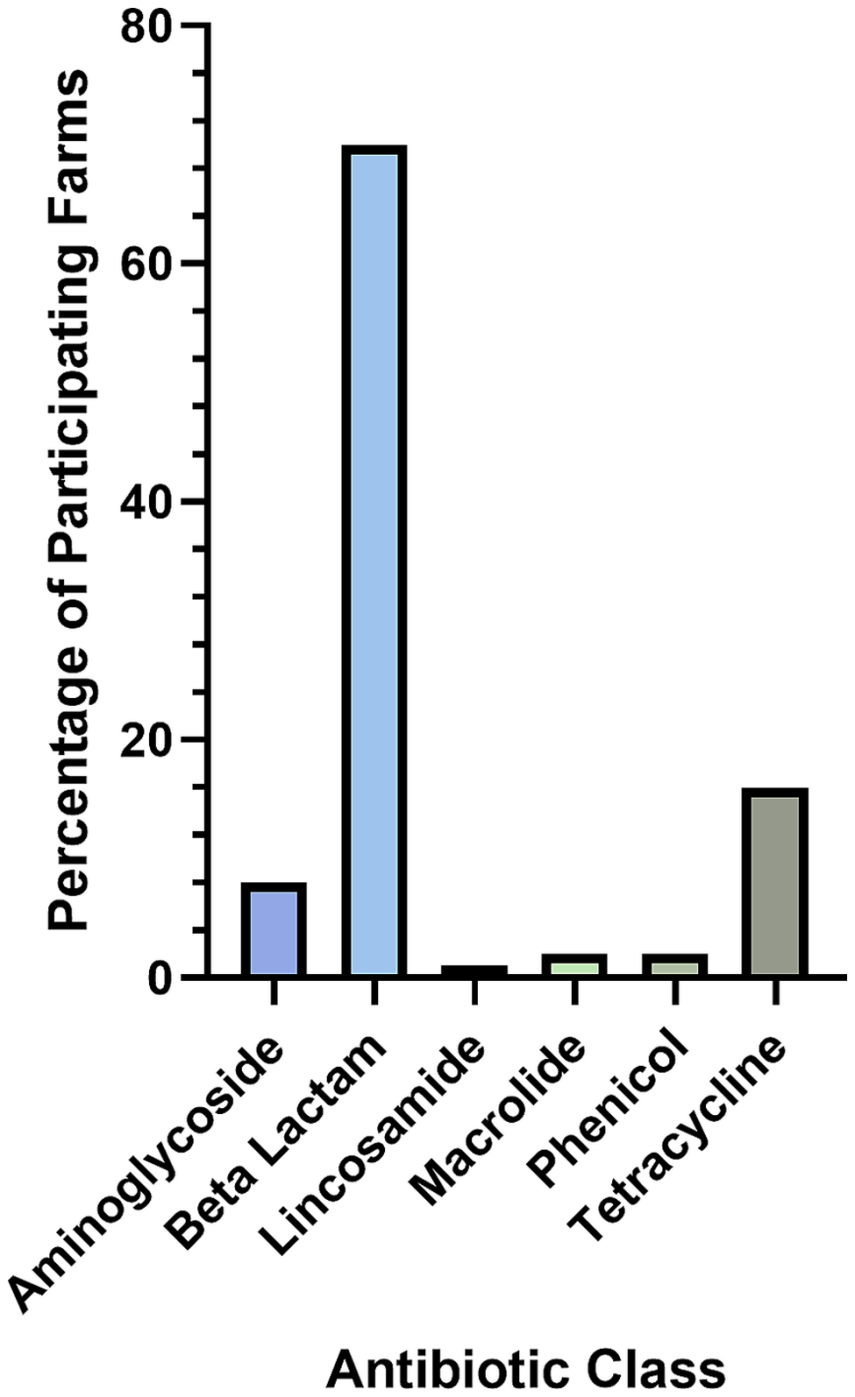
Classes of antibiotics used by farmers to treat NIA cases (nr = 196).

Only 5% of farmers had the bacterial cause of NIA diagnosed by a vet (*N* = 204), with three specifying it as a streptococcal species, and four as *Streptococcus dysgalactiae* (*n* = 7). Antibiotics used by farmers for the treatment of NIA in 2020 lambing season varied, with beta-lactams, including penicillins, the most used class at 70% (Figure 2).

**Figure 3:**
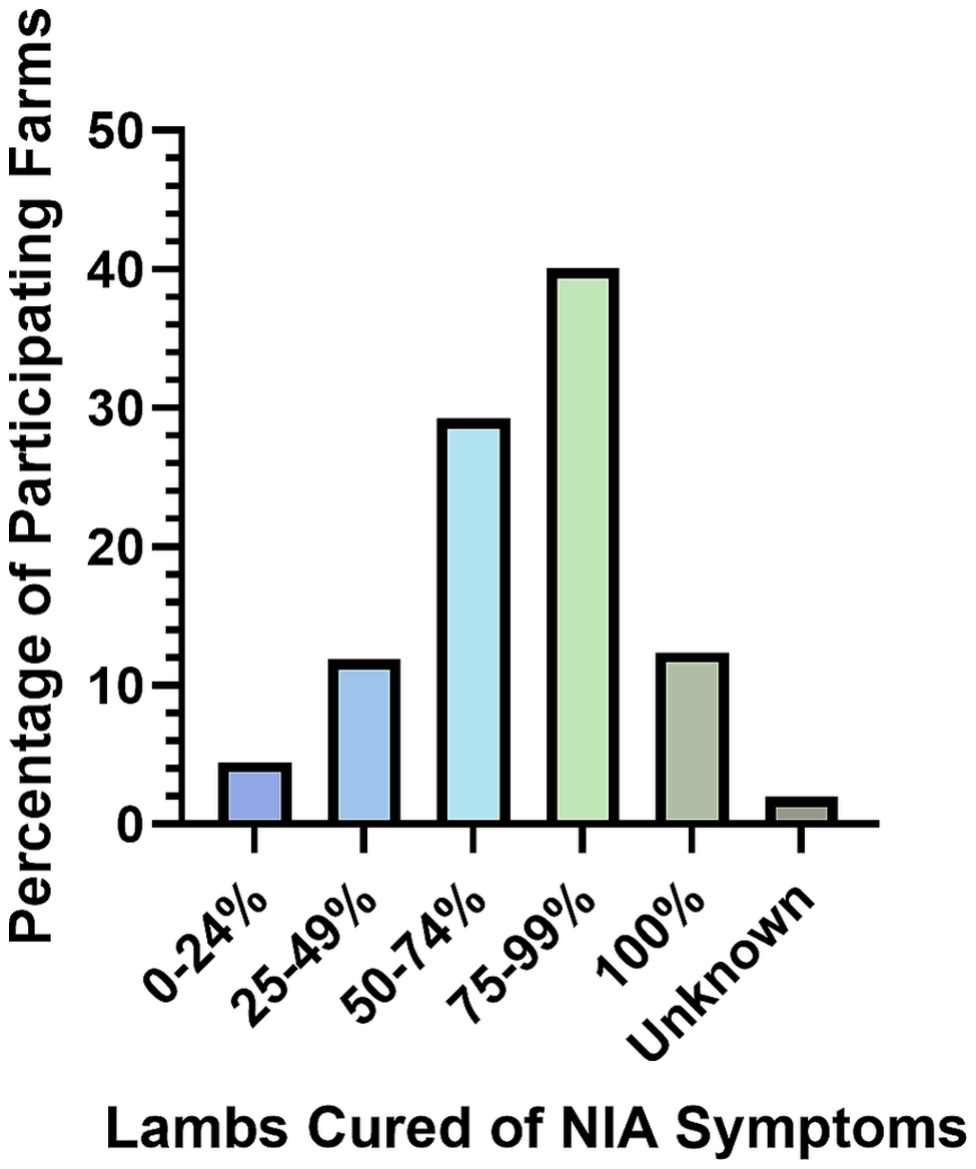
Farmer estimated cure rates for NIA cases (*N* = 202).

Farmers treated lambs for NIA over a median of 5 days (IQR, 1–6); problems with re-catching lambs resulting in inconsistent treatment were mentioned by 4% of farmers (*N* = 194). Of 202 responses, only 12% of farmers reported that 100% of NIA lambs were cured following treatment (Figure 3). Thirty-one farmers listed non-steroidal anti-inflammatory medication in combination with antibiotic treatment (*N* = 197).

#### Prevention of NIA

**Figure 4:**
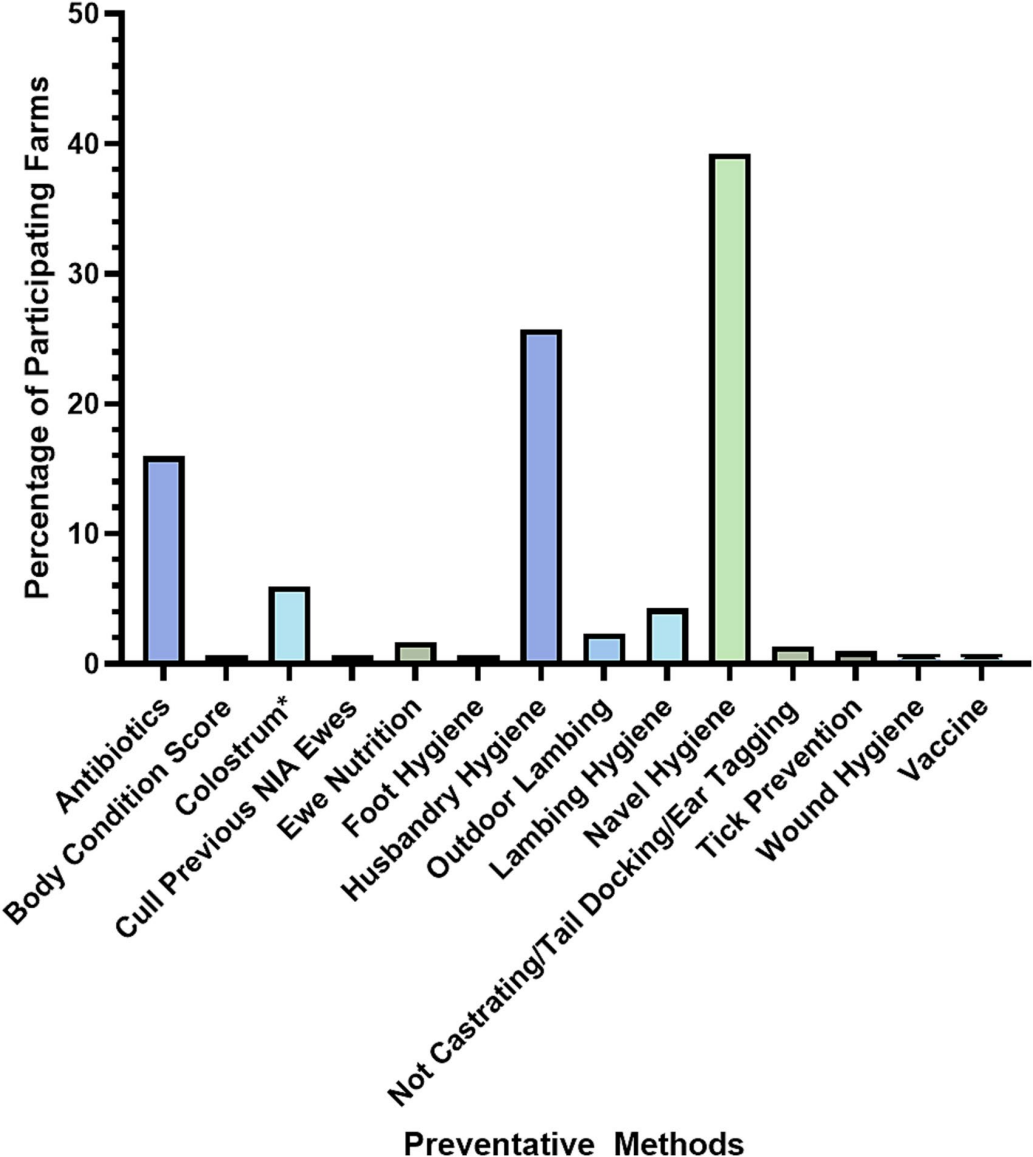
Prevention methods of NIA control employed by farmers (*N* = 213). *Colostrum refers to the management of colostrum intake.

When asked if they used specific preventative measures for NIA, 67% of farmers said yes, compared to 33% saying no (*N* = 321). Farmers that responded ‘yes’ were then asked what preventative methods they used; 16% of the respondents to this question (*N* = 213) cited antibiotics as their prevention method for NIA, while 84% of these respondents listed other measures of prevention (*N* = 213). Common themes of other preventative methods were determined by giving the farmer a free text answer option. Themes identified were hygiene practices, in particular lamb navel hygiene, with 39% of farmers listing this as a prevention method and ‘husbandry hygiene’, with 26% of farmers citing this as a preventive method for NIA (*N* = 213; Figure 4).

**Figure 5:**
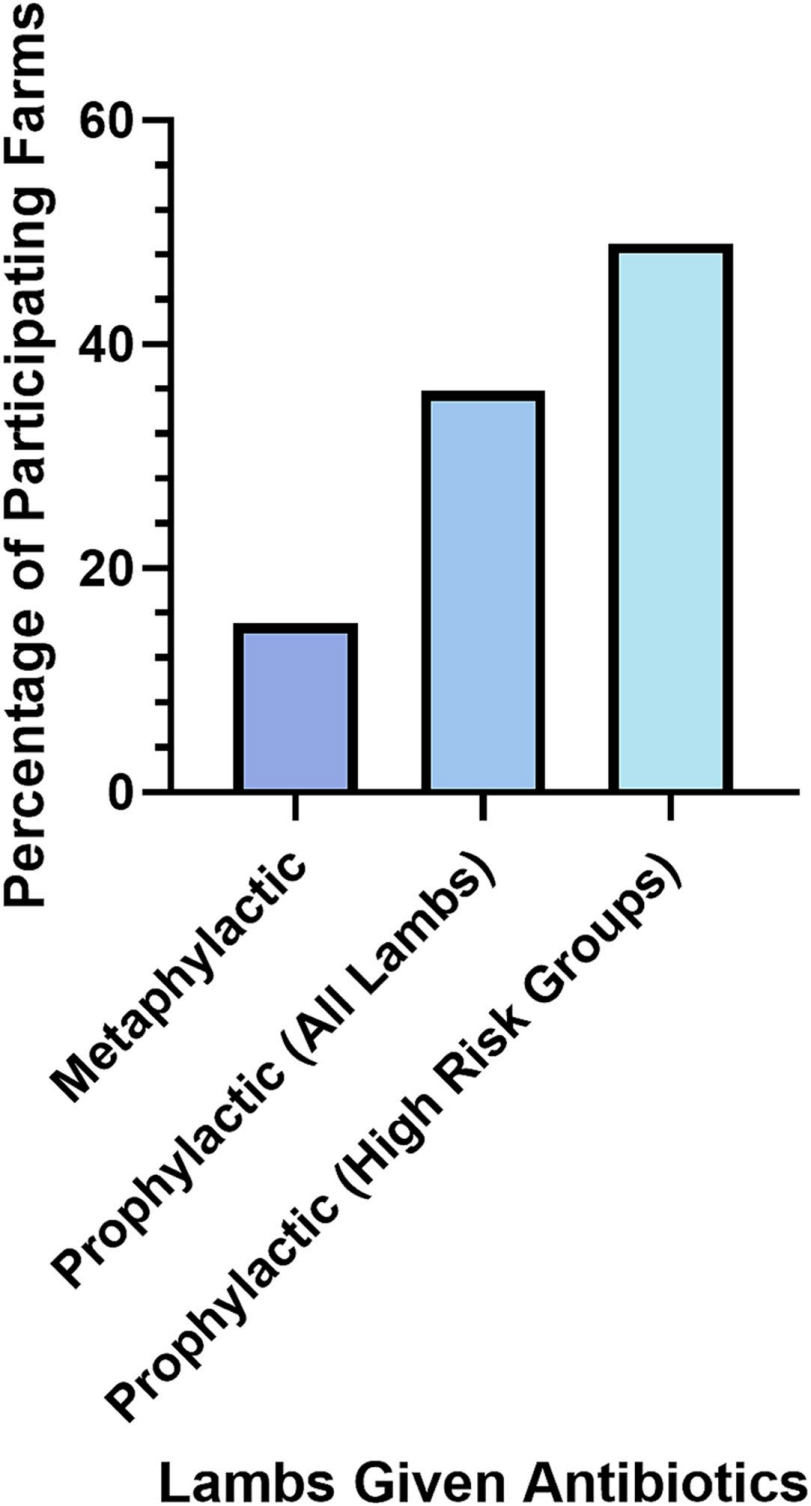
Lambs given antibiotics as a preventative method for NIA as reported by participant farmers (*N*=170).

Antibiotics were used as a preventative measure for NIA by 16% of farmers (*N* = 213). In an additional question, 36% of farmers said they gave all lambs prophylactic antibiotics routinely, regardless of NIA outbreaks (*N* = 50; Figure 5).

### Farmer reported NIA vaccine uptake

The survey was used to determine if there was a demand for a NIA vaccine among UK sheep farmers. Forty-nine percent of farmers said they would be likely or high likely to use a NIA vaccine on their flock. Fifty-one percent said they would be unlikely or highly unlikely to use a vaccine in their flock (*N* = 321).

### Description of lambing management practiced by UK sheep farmers in lambing season 2019–2020

The data is summarised in Supplementary material 6, Table C.

#### Outdoor lambing flocks

Fifty-two percent of farmers lambed outdoors (*N* = 322) at some point during the 2020 lambing season, with 26% lambing completely outdoors and 26% using a mix of indoor and outdoor lambing. Of farmers that lambed outdoors, 73% did not provide additional shelter to ewes lambing outside (*N* = 162). Most farmers moved their newly lambed ewes and lambs to a different field from which they lamb within 1–3 days of lambing (27%), compared with within 24 h (19%), 4–7 days (11%), and over a week (7%). Having a set stock that was not moved on to an alternative location following lambing was described by 16% of farmers, with 20% of farmers describing the move on time as being varied (*N* = 159). Reasons for variation included factors such as ‘weather’ (50%), ‘lamb health’ (25%), ‘ewe’ issues including age and mismothering (12%), ‘grass availability’ (17%), and ‘space’ in fields (13%). Four farmers also noted a difference in move on time for twins/singles/triplets bearing ewes.

#### Indoor lambing flocks

There were 147 farmers who lambed entirely indoors in the 2020 lambing season (47%), with 25% utilising a mix of indoor and outdoor lambing (*N* = 309). Therefore, the total farms lambing indoors, either entirely or at some point during lambing, was 223 (72%). Ewes were housed for a median of 4 weeks before lambing (IQR, 2–6; *N* = 205).

#### Pen management

Questions were asked relating to lambing pen management and hygiene.

Seventy-two percent of farmers said they cleaned group pens prior to lambing (*N* = 231). Separate individual mothering pens were used by most farmers; 94% yes, 5% sometimes, 0.4% no (*N* = 234), with a median of 40 mothering pens being available during lambing (IQR, 20–70; *N* = 211). Bedding used in mothering pens was mainly straw (94%), with shavings being used by 5% of farmers, and 1% of farmers using another, unspecified substrate as bedding (*N* = 233). When asked how often mothering pens were cleaned, 38% of farmers topped up fresh bedding between every ewe and 34% cleaned out bedding between every ewe, 13% said they cleaned when soiled/visibly dirty, 1% of farmers cleaned pens approximately once a week and 12% of farmers cleaned out pens at the end of the season. In addition, 30% of farmers selected more than one option (*N* = 233). Other cleaning methods for mothering pens were described by 1% of farmers. When asked if mothering pens are regularly disinfected, 70% of farmers said yes, compared to 30% saying no (*N* = 231). Of the 28 disinfectants described by farmers, 43% of farmers used lime, 12% Virkon, 7% Stalosan 6% Sorgene (6%), and 4% Fam 30 (*N* = 167).

When asked how long ewes and lambs remained in individual mothering pens, 35% of farmers described 1–3 days, with 27% saying 24 h and only 2% saying 4–7 days (*N* = 232). However, most farmers said the time ewes and lambs spent in mothering pens varied (36%). Reasons for variation included lamb health (35%), ewe health (18%), mothering ability (18%), weather (16%), and whether lambs were singles, doubles, or triples (11%). Also mentioned was space (1%) and colostrum intake (1%; *N* = 102).

Nursery pens were used by 85% of farmers (56% yes and 29% sometimes), compared to 15% of farmers who did not use nursery pens before turnout (*N* = 233). Straw was used as bedding in nursery pens by 99% of farmers, with only 1% opting for shavings (*N* = 197). Topping up fresh bedding between every group of ewes was selected by 43% of farmers when asked how frequently they cleaned nursery pens, with 26% saying they cleaned nursery pens at the end of the lambing season. Cleaning nursery pens when it was required, when dirty/soiled, was selected by 14% of farmers, followed by cleaning nursery pens between every group of ewes (6%), cleaning nursery pens everyday (3%), every couple of days (2%), and once a week (2%; *N* = 283). More than one option was selected by 74 farmers (38%).Other frequencies of cleaning nursery pens were described by 3% of farmers. Most farmers said they did not regularly disinfect nursey pens; 61% no to 39% yes (*N* = 195). Of those that disinfected nursery pens, the vast majority (53%) used lime, with Virkon the second most used disinfectant (17%; *N* = 77). When asked how long ewes and lambs remained in nursery pens, 44% of farmers described 1–3 days, followed by 24 h (13%), 4–7 days (9%), and over a week (6%; *N* = 198). Variation in time spent in nursery pens was described by 28% of farmers.

In total, most lambs and ewes remained indoors for 1–3 days before turnout onto pasture (45%), with 17% being turned out after 4–7 days, 10% after a week, and 10% after 24 h. The time before turnout varied for 18% of farmers (*N* = 233).

#### Body condition scoring

Body condition scoring ewes prior to lambing was practiced by 55% of farmers, compared with 45% who do not (*N* = 319). Of those that body condition scored ewes, the median body condition score target was 3, with a minimum of 2 and a maximum of 4 being reported (IQR, 3–4; *N* = 170).

#### Colostrum management

When asked about their colostrum management, 85% of farmers said they monitored ewes and lambs for colostrum intake, 9% monitored sometimes and 6% did not monitor for colostrum (*N* = 319). The subsequent question explored how this was done by the farmer. Checking lambs for fullness of stomach was the most common way of monitoring colostrum intake (48%).This was followed by checking for the presence of colostrum in the ewe’s udder (25%), observing lambs suckling the ewe (24%), other methods (2%), and blood test monitoring of colostrum transfer via a vet (1%).Other methods for colostrum monitoring described by farmers included manually turning over ewes to enable lamb suckling (29%) and using a refractometer (29%; *N* = 8).

The majority of farmers said they supplemented colostrum (73%; *N* = 321), with lambs who do not suckle within 6 h of birth (26%), triples (25%), those born to old/poor body condition ewes (23%), and lambs who do not immediately suckle (15%) being the most common groups receiving supplemental colostrum (*N* = 540). Twins/doubles were the group least supplemented (1%), with all lambs (2%) and those who do not suckle within 24 h (4%) following closely. Other groups given supplementary colostrum were described by 23 farmers; lambs of ewes with insufficient colostrum were supplemented by 53% of farmers. Also listed included struggling lambs, e.g., hypothermic, difficult labours, mismothered (33%), orphans (10%), and all lambs (3%).

The predominant source of supplementary colostrum was commercial (also known as artificial; 40%), followed by ewe’s own colostrum (26%) and fresh colostrum from another ewe (20%). Cow colostrum was least used (6%), with frozen colostrum from another ewe following closely (8%); one farmer used goat colostrum (*N* = 515). Using more than one source of supplementary colostrum was described by 68% of farmers. The amount of supplemented colostrum given to lambs ranged from 5 mL to 700 mL, with a median of 120 mL (IQR, 75–150; *N* = 217).

Most farmers said they regularly cleaned stomach tubes (97%; *N* = 232), with between each lamb the most common frequency of cleaning (64%). Stomach tubes were cleaned everyday by 19% of farmers, followed by 15% of farmers saying they cleaned stomach tubes between each ewe. Only 2% of farmers said they cleaned stomach tubes weekly, with 1% cleaning after dosing sick lambs (*N* = 238). Bottles and teats were cleaned by 99% of farmers (nr = 231), with 59% cleaning between each lamb. 39% cleaned feeding equipment daily, followed by 1 and 2% that cleaned only after feeding sick lambs and weekly, respectively (*N* = 246).

#### Staff management

A median of two staff members were involved with lambing on farm featured in this study, with a minimum of one and a maximum of six (IQR, 2–3; *N* = 319). Gloves were reported to be worn when lambing by 40% of farmers, with 32% only wearing them sometimes and 28% not wearing gloves at all (*N* = 321). Staff washed their hands between lambing ewes on 73% of farms, with 20% only washing hands sometimes and 7% of farms reported staff did not wash their hands between lambing ewes (nr = 321). When washing their hands, staff used soap and water (61%), while disinfectant was used by staff of 24% of farms (*N* = 370). A combination of hand washing techniques were described by 21% of farmers (*n* = 79).

#### Lambing hygiene practices

Lastly, questions regarding hygiene measures surrounding lambing practices were asked. Ewes were routinely ‘dagged’ (removal of mud and faeces from wool on rear) on 40% of the study farms, with 30% only dagging if dirty and 30% not dagging routinely (*N* = 319). Head ropes were reported to be routinely cleaned between each use by 84% of farmers, with 9 and 7% saying they are cleaning daily and weekly, respectively (*N* = 305).

Lambs’ navels were treated by 89% of farms in this study (*N* = 318), with 69% treating them once and 31% treating navels twice. When asked when lambs navels are treated, most farmers (79%) treated navels within 2 h of birth. Treatment of navels within 6 h was reported by 14% of farmers, with only 5% describing treating lambs within 12 h (*N* = 322). Other answers given via open text response included second treatments at 48 h (*n* = 1) and at tail docking (farmer = 1), only treating navels if lambs were lambed indoors (*n* = 1), with six farmers specifying they treated navels at birth (*N* = 8).Iodine was the most common product used, with 92% of farmers saying they used it to treat lambs’ navels; only 1% of farmers (*n* = 3) said they used topical antibiotics and 7% of farmers said they used another disinfectant (*N* = 284). Other products described by farmers for the treatment of lambs’ navels included surgical spirit, either alone or mixed with iodine (29%), copper sulphate (21%), and Bactakill (Osmonds, UK; 26%; *N* = 28). Spray was the most common method of navel treatment administration, with 56% of farmers opting for this method compared to 42% of farms dipping lambs (*N* = 284). Spraying and dipping was described by four farmers, and two farmers soaked the navel from a bottle (*N* = 6).

Not ear tagging lambs was described by 55% of farmers (*N* = 316). Of farmers that did ear tag (45%), lambs were tagged at median of 2 days old (IQR, 1–7; nr = 140;). Cleaning ear tagging equipment and tags before using was described by 51% of farmers (*N* = 148). Castration was performed by 66% of farmers, compared to 34% who said they did not castrate (*N* = 316). Lambs were castrated between 1 day and 1 week old (58%), with 33% being castrated within 24 h of birth. This is followed by 6 and 3% of lambs being castrated who were by older than 1 week and within 6 h of birth, respectively (*N* = 212). Castration equipment was not cleaned before use by 67% of farmers (*N* = 212). Of the 83% of farms that tail docked lambs (*N* = 317), most lambs were tail docked between 1 day and 1 week old at the time of docking (57%). Tail docking lambs within 24 h of birth was described by 33% of farmers, followed by within 6 h of birth (4%) and older than 1 week (6%; *N* = 267). Tail docking equipment was not cleaned prior to use by 71% of farms, compared with 29% who did routinely clean tail docking equipment, including rings, before use (*N* = 268).

### Univariable and multivariable risk factor analysis for NIA occurrence

Univariable and multivariable analysis was undertaken to investigate associations between predicator variables and the outcome variable. The outcome variable was “farmer reported presence of NIA in the year 2020,” “yes/no.” Analyses were conducted for each of the three sub-sets of the data: ‘Indoor Lambing Flocks’ (*N* = 234), ‘Outdoor Lambing Flocks’ (*N* = 164), and ‘All Flocks’ (*N* = 321).

#### Indoor lambing flocks

Univariable analysis of the indoor lambing flock dataset (*N* = 234) was conducted on 52 relevant variables (Supplementary material 3) and following screening, 17 variables were offered to the model. Of the 234 farms selected for the Indoor Lamb Flock dataset, 66% reported NIA in 2020. Three variables remained in the final multivariable model (Table 2). There was a positive linear relationship between NIA occurrence and the number of lambs born alive; the more lambs born alive, the greater the odds of NIA (number of lambs born alive 501–1,000; OR = 4.1; 95% CI, 1.6–10.7. Number of lambs born alive >1,000; OR, 14.0; 95% CI, 4.0–48.9).

**Table 2:** Final multivariable model for risk factors associated with NIA occurrence in indoor lambing response farms, during the 2020 lambing season.

Variable	Number of farms with NIA	Percentage of farms with NIA	Odds ratio	95% CI	*p* value
Flock type (*N* = 222)	**150**	**67.6%**			**0.037**
Lowland (reference) (*n* = 147)	90	61.2%			
Upland (*n* = 71)	57	80.3%	3.0	1.3, 6.8	0.011
Mountain (*n* = 4)	3	75.0%	0.7	0.02, 21.6	0.824
Lambs born alive (*N* = 219)	**142**	**64.8%**			**<0.001**
1–160 (reference) (*n* = 54)	22	40.7%			
161–500 (*n* = 60)	36	60.0%	2.6	1.0, 6.4	0.043
501–1,000 (*n* = 53)	39	73.6%	4.1	1.6, 10.7	0.004
1,001+ (*n* = 52)	45	86.5%	14.0	4.0, 48.9	<0.001
Washing hands (*N* = 234)	**155**	**66.2%**			**0.018**
Yes (reference) (*n =* 178)	108	60.7%			
No or sometimes (*n* = 56)	11	91.7%	3.6	1.2, 10.6	0.018

The overarching question values are marked in bold, with the responses directly following.

Upland farms reported greater odds of NIA than lowland and mountain farms (OR = 3.0, 95% CI, 1.3–6.8). The odds of NIA were higher on farms that did not practice regular hand hygiene (OR = 3.6, 95% CI, 1.2–10.6). Variance inflation factors were acceptable, ranging between 1.02–1.49. The model was shown to have acceptable fit according to the Hosmer-Lemeshow goodness-of-fit test, at *p* = 0.528.

#### Outdoor lambing flocks

Univariable analysis of the outdoor lambing flock dataset (*N* = 164) was conducted on 40 relevant variables (Supplementary material 4) and following screening, nine were offered to the model. Of farms in the Outdoor Lambing Flock dataset, 65% reported NIA cases in 2020. Of the nine variables put forward for multivariable analysis, four variables were included in the final model output (Table 3). A positive linear relationship was observed between the number of ewes lambed and odds of NIA occurrence on response farms (>600 ewes; OR = 34.7, 95% CI, 6.6–182.7). Using antibiotics as a preventative measure was associated with a reduced odds of NIA (OR, 0.1; 95% CI, 0.01–0.4). Not providing outdoor shelter was associated with higher odds of NIA occurrence (OR = 3.0. 95% CI, 1.2–7.8). Not cleaning ear tags and ear tagging equipment was shown to be associated with increased odds of NIA occurrence on outdoor lambing farms (OR = 5.7, 95% CI, 1.5–21.4).Variance inflation factors were acceptable, ranging between 1.06–1.88. The model was shown to have acceptable fit according to the Hosmer-Lemeshow goodness-of-fit test, at *p* = 0.877.

**Table 3:** Final multivariable model for risk factors associated with NIA occurrence in outdoor lambing farms, during the 2020 lambing season.

Variable	Number of farms with NIA	Percentage of farms with NIA	Odds ratio	95% CI	*p* Value
No. ewes lambed (*N* = 154)	**100**	**64.9%**			**0.001**
1–100 (reference) (*n* = 39)	13	33.3%			
101–300 (*n* = 29)	18	62.1%	2.2	0.6, 7.2	0.213
301–600 (*n* = 42)	29	69.1%	3.3	1.1, 10.2	0.039
601+ (*n* = 44)	40	90.9%	34.7	6.6, 182.7	<0.001
Antibiotics as preventative method (*N* = 163)	**107**	**65.6%**			**0.001**
No (reference) (*n* = 80)	61	76.3%			
Yes (*n* = 20)	12	60.0%	0.1	0.01, 0.4	0.004
Does not use preventative measures (*n* = 63)	34	54.0%	0.2	0.1, 0.5	0.001
Outdoor shelter (*n* = 160)	**104**	**65.0%**			**0.022**
Yes (reference) (*n* = 44)	16	36.4%			
No (*n* = 116)	88	75.9%	3.0	1.2, 7.8	0.022
Cleaning ear tags (*N* = 164)	**107**	**65.2%**			**0.022**
Yes (reference) (*n* = 39)	17	43.6%			
No (*n* = 38)	25	65.8%	5.7	1.5, 21.4	0.010
Does not ear tag (*n* = 87)	65	74.7%	3.8	1.3, 11.4	0.018

The overarching question values are marked in bold, with the responses directly following.

#### All flocks

Univariable analysis of the all-flock dataset (*n* = 321) was conducted on 38 relevant variables (Supplementary material 5) and following screening, 11 variables were offered to the model (Table 4). Of the 321 farms in our All-Flock dataset, 64% reported NIA in 2020. Of the 11 variables included for multivariable analysis, one variable was included in the final model output (Table 4). A positive linear relationship was once again observed between the number of ewes lambed and NIA occurrence on response farms; the odds of having NIA cases increased with flock size (301–600 ewes; OR = 3.9, 95% CI 1.9–8.0. >600 ewes; OR = 13.7, 95% CI, 5.4–34.4). Variance inflation factors were acceptable, ranging between 1.03–1.43. The model was shown to have acceptable fit according to the Hosmer-Lemeshow goodness-of-fit test, at *p* = 0.905.

**Table 4:** Final multivariable model for risk factors associated with NIA occurrence in All Flocks, during the 2020 lambing season.

Variable	Number of farms with NIA	Percentage of farms with NIA	Odds ratio	95% CI	*p* value
No. ewes lambed (*N* = 307)	**195**	**63.5%**			**<0.001**
1–100 (reference) (*n* = 80)	30	37.5%			
101–300 (*n* = 74)	43	58.1%	1.7	0.9, 3.5	0.126
301–600 (*n* = 79)	58	73.4%	3.9	1.9, 8.0	<0.001
601+ (*n* = 74)	64	86.5%	13.7	5.4, 34.4	<0.001

The overarching question values are marked in bold, with the responses directly following.

## Discussion

NIA is considered a common disease and a key driver of antibiotic use on UK sheep farms (9). To our knowledge, this study is the largest ever prevalence and farm risk factor study of NIA conducted in the UK. In 2020, 64% of farmers reported experiencing NIA, with the disease affecting indoor, mixed, and outdoor lambing flocks. The median within flock incidence during the 2020 lambing season was 1.4% (IQR, 0.8–2.6; 95% CI, 1.2–1.6) which is in keeping with previously reported UK within farm incidence of ~2% from a study of 36 sheep flocks (4).

The farmer reported practices for treatment and prevention of NIA provide a useful insight for veterinarians and the sheep industry on what aspects of NIA case management to emphasise when communicating with individual farms. Firstly, this study highlights a substantial lack of veterinary diagnosis for NIA cases, with only 5% of farms receiving a specific bacterial cause by their veterinarian. This is important, as although NIA is most often caused by SDSD, it can be caused by other agents such as *Staphylococcus aureus* and *Erysipelothrix rhusiopathiae* which have different risk factors for infection that need to be addressed to prevent cases (1, 19, 20). Of greater importance for veterinary investigation of cases is ensuring the lambs are being treated correctly; 25% of farms reported using antibiotics that are routinely reported to be ineffective against SDSD (tetracycline and aminoglycoside antibiotics) (21) to treat NIA cases. In addition, 49% of farmers reported NIA case lambs were treated for less than the minimum recommended period of 5 days (*N* = 194) and only 52% of farmers consider treatments to be highly effective (more than 75% of lambs cured; *N* = 202). This suggests there is an urgent need for accurate diagnoses of NIA cases based on bacterial culture, with antimicrobial susceptibility testing, compliance with veterinary designed treatment protocols, and an emphasis on treating NIA cases as soon as they are observed (22).

In the limited epidemiological literature on SDSD NIA (4, 12), the evidence indicates that the ewe may be a source of SDSD, which is then transmitted to lambs via the lambing environment. In addition, as this study shows that most (70%) NIA cases occur within the first 2 weeks of life, and that NIA is observed in indoor and outdoor lambing flocks, then it important to gain a picture of current farm hygiene practices in both environments for the lambs during this risk period. For indoor lambing flocks, 94% of farmers used straw as a bedding, with 34% following the guidance that individual pens should be cleaned out of bedding between each ewe and lamb (4). Eighty-five percent of farmers used group nursery pens before turnout of lambs; 99% of these were also straw bedded, however most farmers (59%) only cleaned these out at the end of the lambing season. To improve guidance on farm environmental hygiene practices, further studies are needed to confirm if SDSD can be found in indoor and outdoor lambing environments, particularly in straw and soil. Additionally, how long SDSD survives in these substrates and what disinfectants would be effective in their control, are important factors to consider for on farm NIA control and thus, is an important area of future NIA research.

Failure of passive transfer of maternal immunoglobulins (FPT) is a recognised risk factor for lamb neonatal morbidity and mortality (15), although, its role in NIA is currently unknown. However, it is encouraging that 87% of farmers reported monitoring colostrum intake, 73% said they used colostrum supplementation, and 97% reported regularly cleaned feeding equipment. However, it is important to consider social desirability bias, as sector guidance heavily emphasises the importance of a robust colostrum policy. While data anonymity and confidentiality were assured at the beginning of the survey, it is possible responses throughout the survey were biased towards believed ‘correct’ responses.

The lamb navel is a hypothesised route of entry of SDSD-NIA (2). Therefore, it was promising to see 89% of farmers reported disinfecting navels, with 31% following guidance to treat navels twice, and 92% using iodine, as recommended (22). Neonatal lamb wounds caused by management practices such as ear tagging, castration, and tail docking have been hypothesised as routes of entry of SDSD infection, with recent studies supporting the potential role of wounds in the pathway to infection (23). In this study, these were common practices in UK sheep flocks; 45% of farmers ear tagged lambs, 66% castrated lambs, and 83% tail docked lambs. Whether these wounds are an important site of bacterial infection to the neonatal lamb is not yet established, however, most farmers did not clean equipment, and 33% castrated and tail docked lambs at less than 24 h old. This is a known risk factor for ‘watery mouth’ disease, gastrointestinal disease caused by *Escherichia coli* (24).

The study found that prophylactic antibiotic use as a preventative measure for NIA is still an issue within the sector; with 11% of farms (*n* = 34) in the study using this approach (*N* = 322). However, 40% of farms who used antibiotics as a preventative measure, still experienced NIA outbreaks within their flocks (*N* = 34). Furthermore, the risk factor analysis found that use of antibiotics to prevent NIA, was only associated with reduced occurrence of NIA at farm level in outdoor lambing flocks.

Given prophylactic antibiotic use was highlighted as a preventative measure by this study, vaccine development offers a key area of future exploration for the sustainable prevention of NIA. Therefore, it is interesting to note that 49% of farmers in this survey would be likely to use such a vaccine should it be developed.

There is a large diversity of farm management practices undertaken in UK flocks at lambing time, consequently many independent variables had to be recorded in this study. To maximise the model accuracy several steps were taken in the analysis to keep the number of variables per outcome to an approximate recommended ratio of 10 data points per variable (25). These were assessing collinearity of variables, combining variables into a score or scale, and excluding variables with minimum effect on outcome based on univariable analysis. In addition, a sample size calculation was conducted which indicated 381 sheep farms would be required for the study (321 usable responses achieved). However, it is acknowledged given the variation in farm management practices that we were trying to capture, a larger sample size would have been beneficial and added strength to the analysis.

It is worth noting that the outcome variable used in the statistical analysis of this study was presence/absence of NIA disease in the flock, and therefore, the findings do not reflect the burden of NIA cases on a farm.

Though the risk factors associated with NIA outbreaks differed between our three models, a narrative of the intensity of lambing being associated with increased odds of disease occurrence was consistently seen. The number of lambs born in indoor lambing flocks, and flock size in outdoor lambing and all flocks were positively associated with NIA. These findings align with previous risk factor studies, where increased flock size and higher scanning percentage were identified as risk factors for NIA in Norway (12). Interestingly, in the Indoor Lambing Flocks model, farm system was associated with odds of NIA, with lowland farms reporting less risk of NIA cases. Given the higher level of intensification typically seen in lowland systems, compared to upland or mountain, this result is surprising.

Except for ear tag hygiene in outdoor lambing flocks’ model and hand hygiene in the indoor lambing flocks’ model, hygiene factors and lamb management practices were not shown to be associated with NIA outbreaks through multivariable analysis. In the outdoor lambing flocks’ model, cleaning ear tags was shown to significantly reduce the odds of NIA cases, supporting current advice for enhanced hygiene around routine lambing management practices, particularly during wound creation. This correlates with previous risk factor analysis conducted in Norway, where ear tags, particularly those with wound infections, were associated with increased odds of NIA. In the indoor lambing flocks’ model, hand washing was significantly associated with reduced odds of NIA cases on participant farms. The act of frequent hand washing and ensuring good hand hygiene presents a simple additional measure that could be implemented during lambing. It is perhaps surprising that a lack of hygiene factors was not associated with NIA across our models. However, as sector guidance heavily emphasises hygiene for the prevention and reduction of NIA, and most farmers reported they did the recommended levels of hygiene, it is possible that this section of our study was too underpowered to detect significant differences.

Given the hypothesis of bacterial accumulation in the environment and previous studies reporting specific areas were highly associated with NIA outbreaks compared to others, it is interesting that providing outdoor lambing ewes with shelter was associated with reduced odds of NIA cases in our Outdoor Lambing Flocks model. Providing shelter allows for the congregation of ewes to a particular area, which if ewes are indeed a part of the chain of infection, suggests that bacterial accumulation would occur, and NIA risk would be expected to increase.

The respondents within this survey may be considered typical of the UK sheep farming industry, as the geographical distribution of farmer responses reflects the known density of sheep holdings in England and Wales (Figure 1). In addition, the respondents represented a range of farm sizes from three to 3,500 breeding ewes, and all major types of sheep farm were represented including hill, lowland, upland, indoor, and outdoor lambing flocks. However, the study results could be impacted by reporting bias, as farmers suffering from NIA outbreaks may be more likely to respond to the survey than farms who do not. It is also worth considering that the data applies to 2020. However, in the absence of national disease prevalence and incidence data, this study can be considered a useful source of information on farmer reported NIA prevalence, current farm management practices for NIA, and risk factors associated with farms affected by NIA in the United Kingdom.

In conclusion, this study identified a farmer reported between flock prevalence of NIA of 64%, and a within flock incidence of 1.4% (IQR, 0.8–2.6; 95% CI, 1.2–1.6) in UK sheep flocks in 2020. NIA is reported in indoor, outdoor, and mixed lambing flocks. The lack of formal veterinary diagnosis, errors in antibiotic selection, and length of treatment are a concern for animal welfare and responsible antibiotic use within the industry. This highlights a clear need for more veterinary involvement on farms at lambing time, particularly in NIA. As seen across all our models, a pattern of increased intensity of lambing was associated with increased odds of NIA occurrence. Factors such as flock size and the number of lambs born were associated with increased odds of NIA on participant farms. Although prophylactic antibiotic use was associated with reduced NIA odds in outdoor lambing flocks only, farms who utilised this prevention method still experienced NIA cases.

## Data Availability

The datasets presented in this study can be found in online repositories. The names of the repository/repositories and accession number(s) can be found at: https://datacat.liverpool.ac.uk/id/eprint/2787.

## Footnotes

^1^ https://www.onlinesurveys.ac.uk/
