# Supplementary material for: A cross-sectional survey of farmer reported prevalence and farm management practices associated with neonatal infectious arthritis (“joint ill”) in lambs, on UK sheep farms
Source: Front Vet Sci. 2024 Dec 23;11:1489751. doi: 10.3389/fvets.2024.1489751 (PMC11701153; doi:10.3389/fvets.2024.1489751)
Supplement: Supplementary file 1 [file Table_1.DOCX]

# Supplementary Material 1: Full Survey

Farm Management Practices Associated with “Joint ill” in Lambs

Information


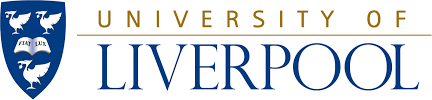


‘Joint ill’ is a disease commonly affecting the joints of young lambs less than one month old. It causes severe pain and lameness, as well as significant financial losses to farms each year. This survey contributes to a project, funded by the University of Liverpool, AHDB Beef and Lamb and in collaboration with The Moredun Research Institute, which aims to find practical solutions for farmers to prevent joint ill. Your participation in this survey will help identify farm management practices which effect the risk of joint ill occurring on farms, and will contribute to new knowledge to help protect flocks.

Please complete this survey if you are a **UK sheep farmer, regardless of whether you do or do not experience joint ill outbreaks.**

This survey should take between 15-20 minutes to complete. To thank you for taking part, you will have the opportunity at the end of the survey to enter a prize draw for an **Apple iPad**, a **Samsung Galaxy Tablet**, or a **Fortnum and Mason Luxury Hamper**.

All data is collected completely anonymously and confidentially, **we will not be able to identify you or your farm from the data collected**. We will hold the data for 10 years on a secure computer system at the University of Liverpool, in accordance with UK General Data Protection Regulations. **You can withdraw your data at any time**

**during completion of the survey, until you press 'Finish' at the end, at which point data will anonymised.** The anonymous results of this survey will be used as part of a research project, be published in scientific journals, and will be used to produce publications for knowledge transfer. By agreeing to take part in this survey, you consent to your data being used in this way. If you have any questions, concerns or feedback about this survey, please contact Louise Jackson at louise.jackson@liverpool.ac.uk


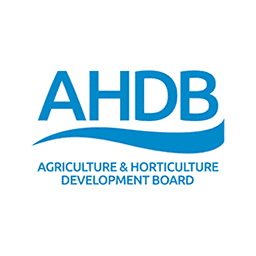
All questions apply to this years 2020 lambing season

1. This section is general information on your whole farm enterprise.

In which county is your farm?


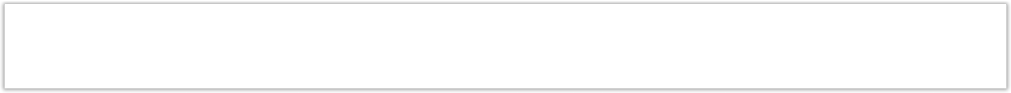


What type of farm do you manage? (Please tick all that apply)


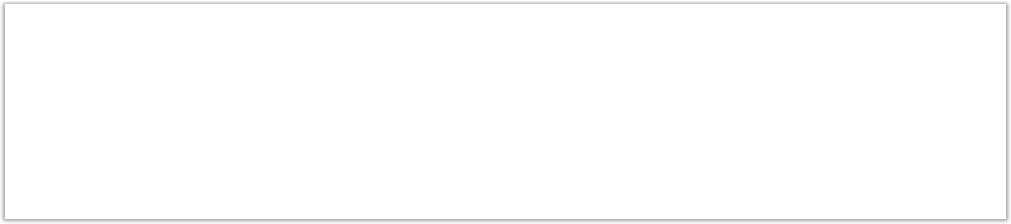


Lowland Upland Organic Mountain

How many **breeding** ewes do you manage in your **whole** farming enterprise?


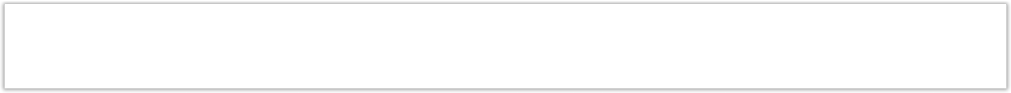


Across your whole farming enterprise, how many hectares of land are used for the sheep flock?


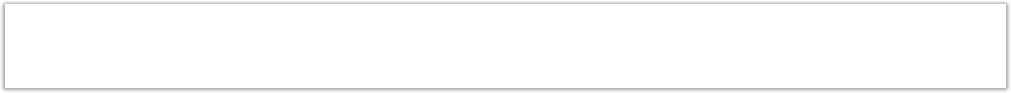


1. This section is general information on this flock.

Many sheep farms manage more than one breeding flock of ewes. If this applies to you, please answer the rest of the survey for your **main (largest) flock**.

Is this flock? (Please tick all that apply)


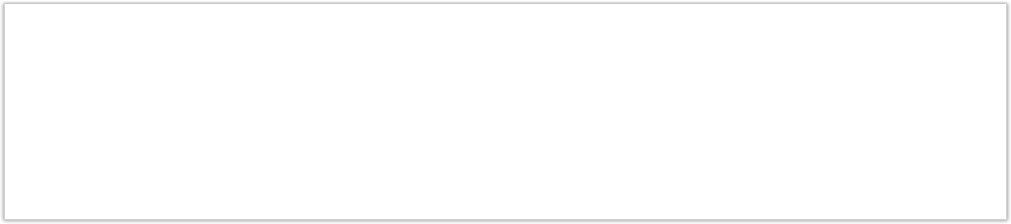


Lowland Upland Organic Mountain

How many **breeding** ewes are in this flock?


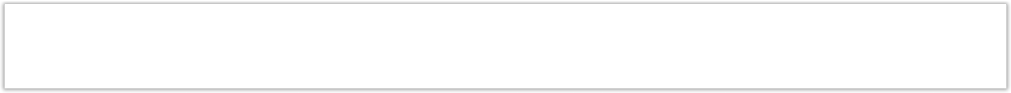


Is this flock pure bred or cross bred?


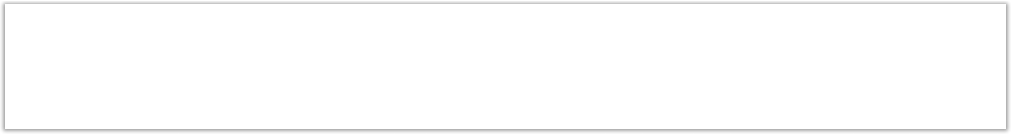

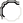

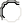


Pure bred Cross bred

What ewe breeds/cross breeds are present?


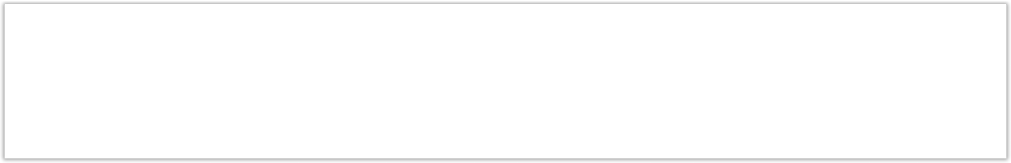


What age category would you describe this flock?


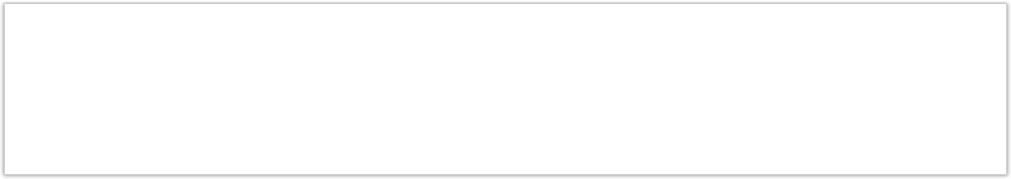

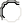

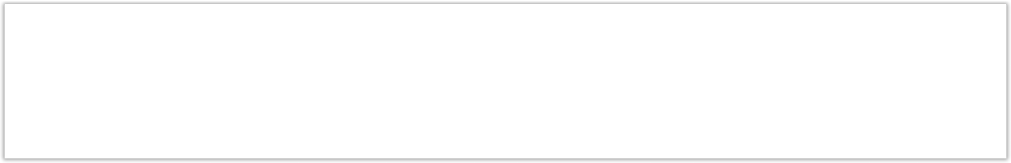

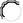


1-2 years old

Majority of ewes more than 5 years old Mixed ages

Where does this flock lamb?


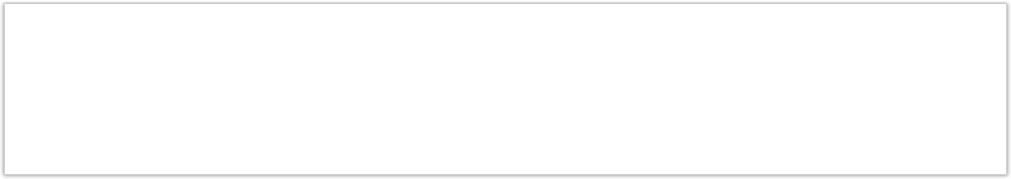

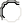

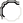

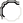


Indoors Outdoors Mixed

In which months does this flock mostly lamb?


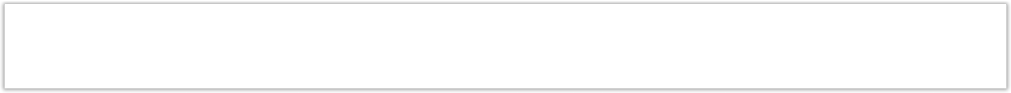


How long does lambing typically last? (in weeks)


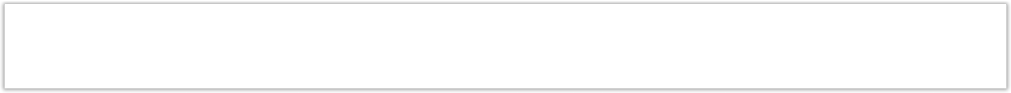


Do you know the scanning percentage of this flock in 2019 mating-2020 lambing season?


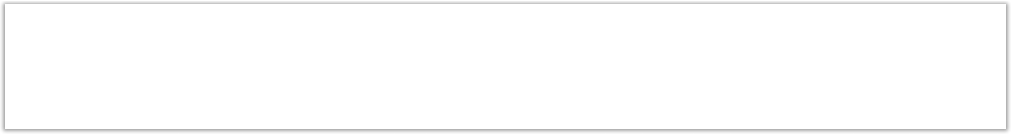

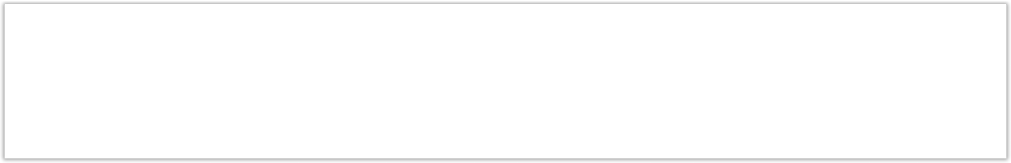

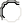


Known Not known

If you selected known, what was the scanning percentage?


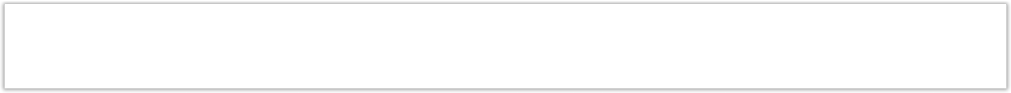


How many ewes lambed in this years 2020 lambing season?


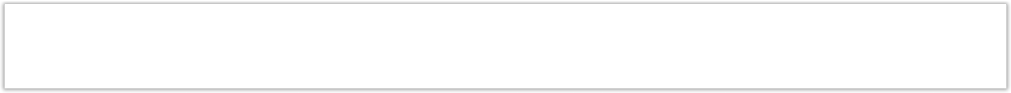


Approximately, how many lambs were born alive for this years 2020 lambing season?


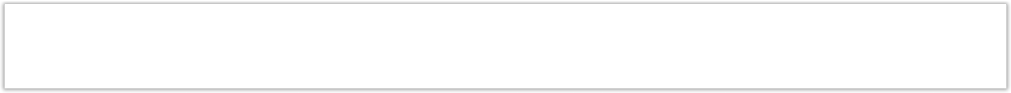


1. This section is specifically about “joint ill”. By “joint ill” we are referring to cases of swollen painful joints in lambs less than one month old

Did you experience "joint ill" in your lambs this year (2020)?


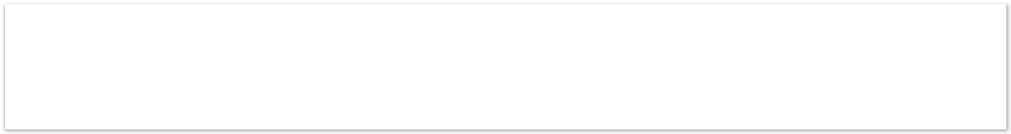

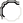

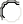


Yes No

1. This section is specifically about “joint ill”. By “joint ill” we are referring to cases of swollen painful joints in lambs less than one month old

How many cases of "joint ill" occured in the **2020** lambing season?


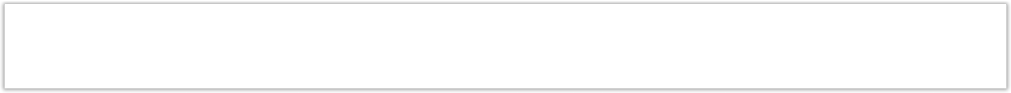


Has a specific bacterial cause been diagnosed by your vet?


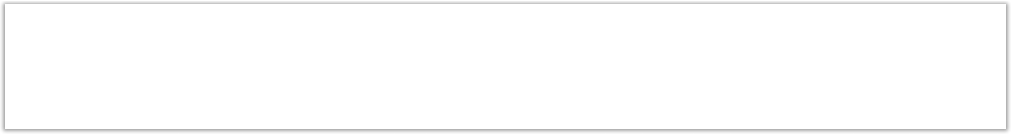

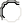

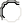


Yes No

If yes, do you know the bacterial cause?


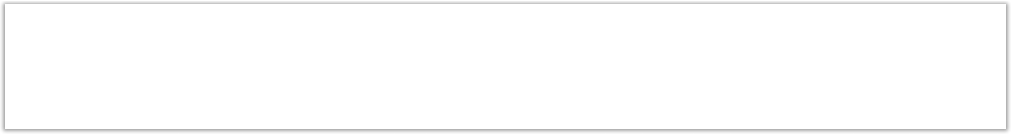

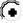

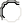


Yes No

If you selected yes, please specify the bacterial cause:


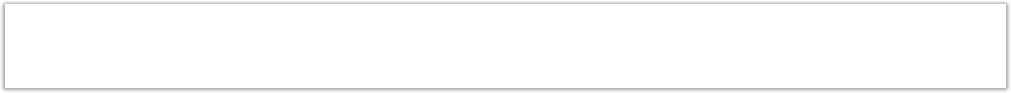


What stage of the lambing period do cases typically occur? (Please tick all that apply)


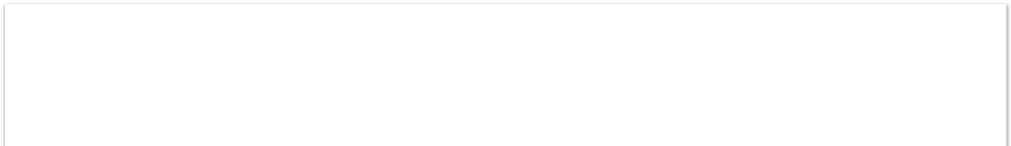


No specific time

Early in lambing period Middle of lambing period


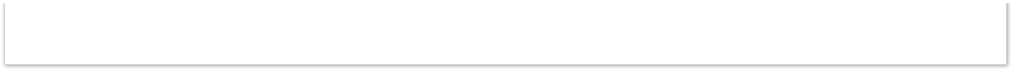


Later in lambing period

Where are joint ill lambs typically born?


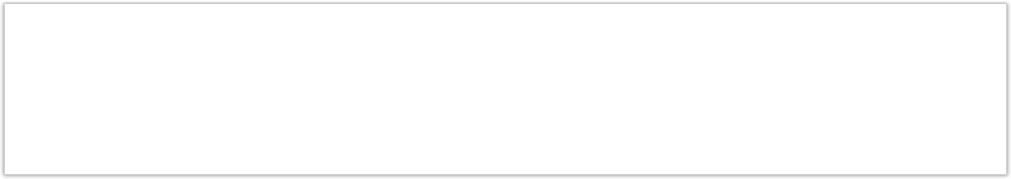

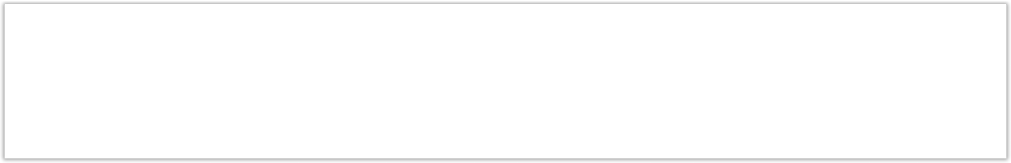

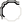

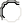


Indoors Outdoors Mixed

At what age do lambs typically develop joint ill? (Please tick all that apply)


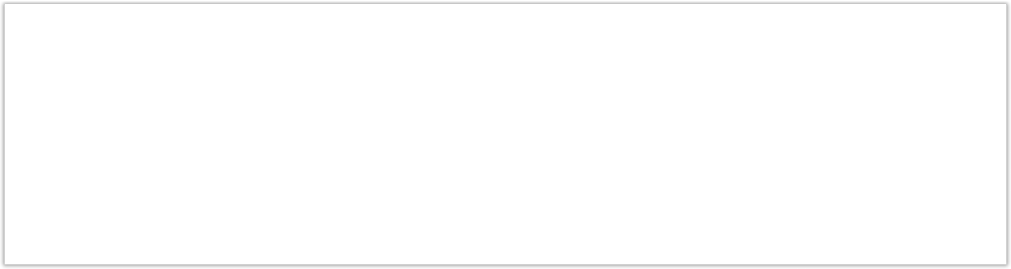


0-3 days old

4-7 days old

8-14 days old

15-28 days old Over 1 month old

What type of lambs are most commonly affected? (Please tick all that apply)


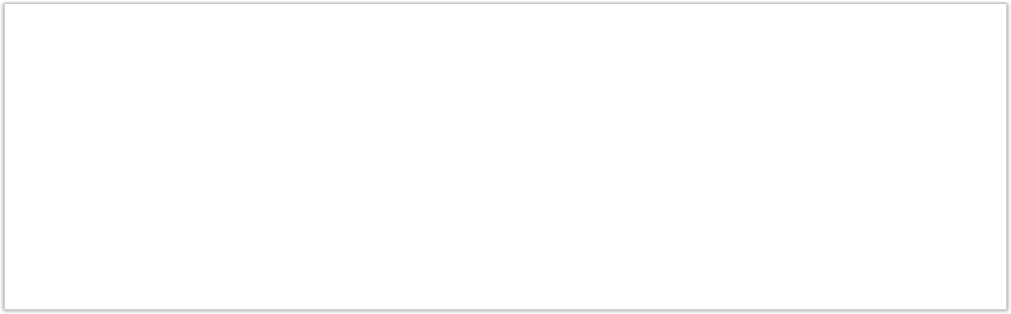


No specific type Singles

Twins Triplets Orphans Other

If you selected other, please specify:


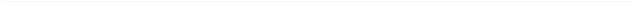


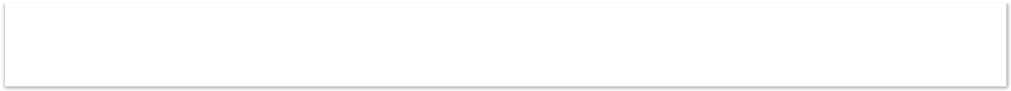


What antibiotic do you use to treat joint ill cases?


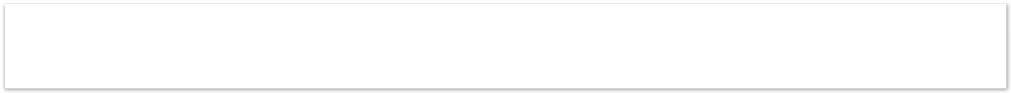


How many days do you treat joint ill cases for?


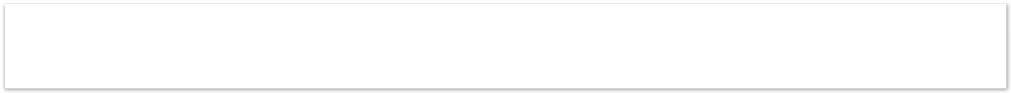


How effective is this treatment?


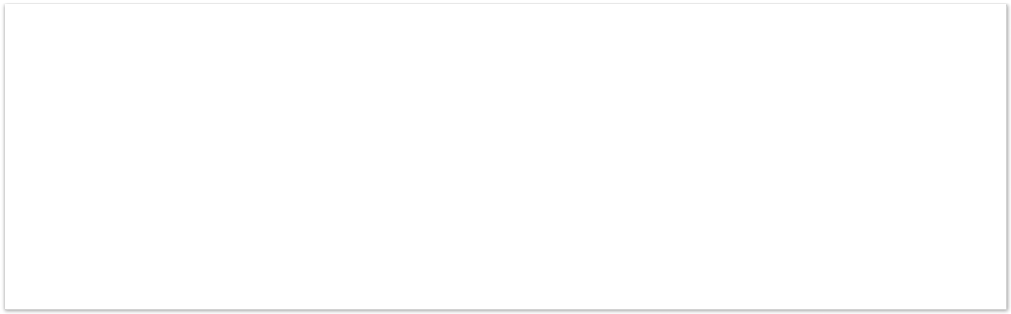

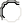

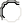

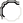

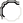

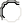

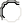


100% cured

75-99% cured

50-74% cured

25-49% cured

0-24% cured Unknown

1. This section is specifically about “joint ill”. By “joint ill” we are referring to cases of swollen painful joints in lambs less than one month old

Do you use any **preventative** measures for joint ill?


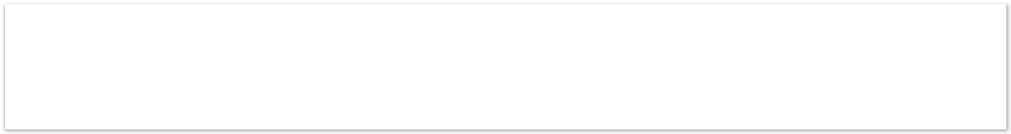

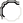

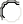


Yes No

1. This section is specifically about “joint ill”. By “joint ill” we are referring to cases of swollen painful joints in lambs less than one month old

What do you use to **prevent** joint ill?


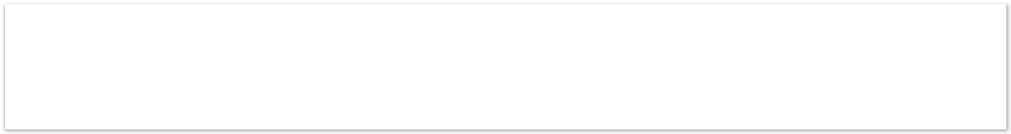

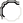

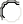


Antibiotics Other

If you selected other, please specify:


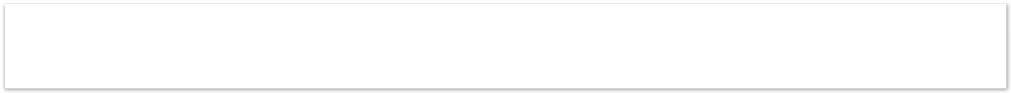


If you use **antibiotics** as a preventative measure, which lambs do you give this to?


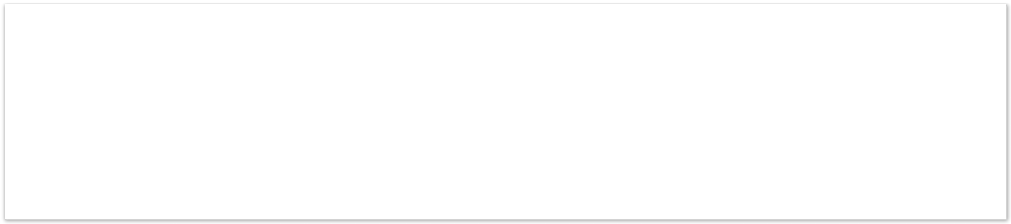

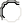

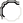

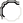

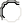


I do not use antibiotics

All lambs given antibiotics routinely whether or not joint ill is present in flock All lambs born after joint ill cases start to occur in the flock

Only specific high-risk groups (e.g. orphans, triplets)

In your experience, what factors do you believe contribute to joint ill occurrence? (Please tick all that apply)

Age of ewe - old ewes Age of ewe - young


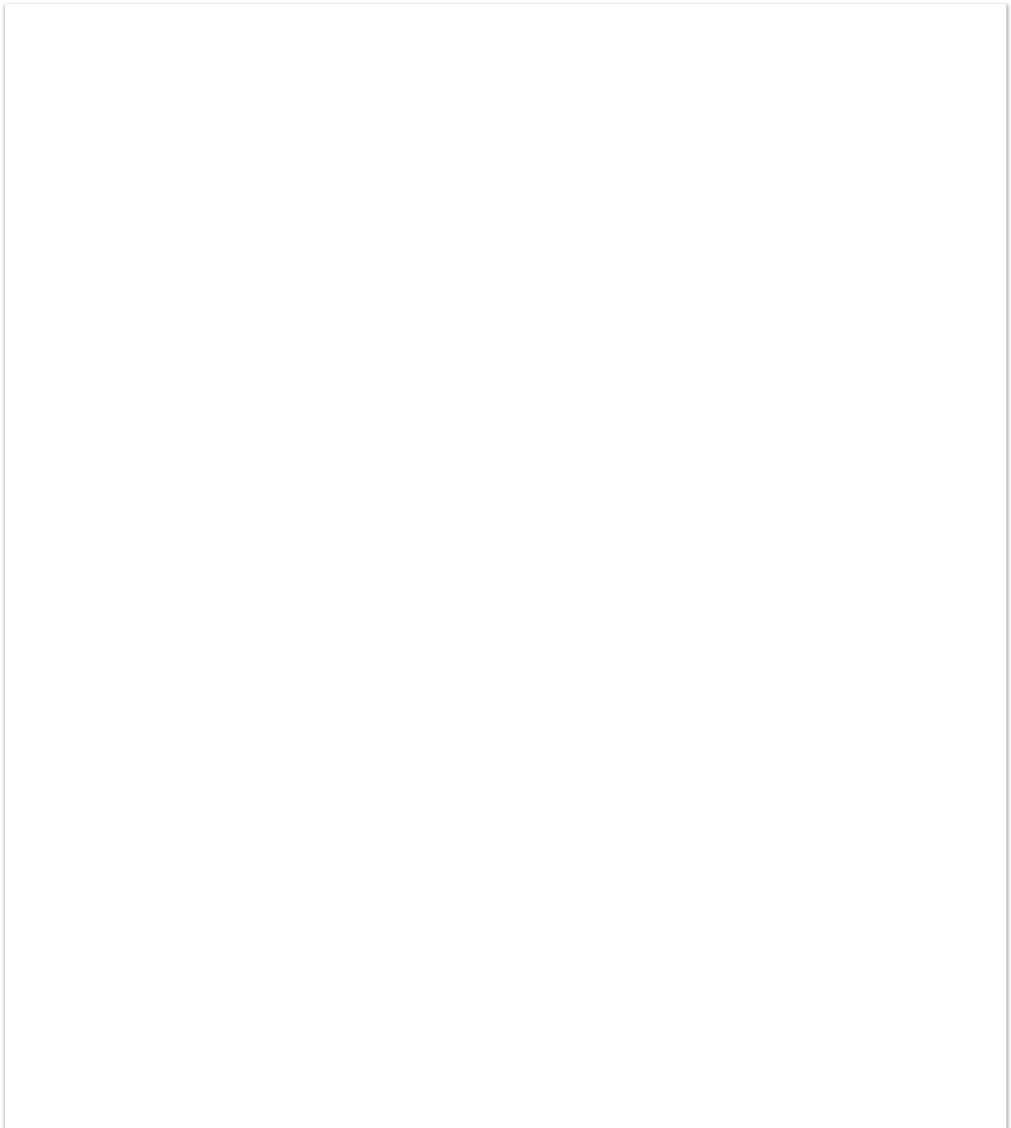


ewes

Poor body condition score ewes

Trace element deficiencies

Cleanliness of ewes Mastitis in ewes

Inadequate colostrum intake

Inadequate navel disinfection

Hand hygiene of staff when lambing

Infrequent/no disinfection of feeding equipment

Castrating lambs Tail docking lambs

Ear tagging lambs Infrequent/no disinfection of tagging, castration and tail docking equipment

Hygiene of pre- lambing pens

Hygiene of separate individual mothering pens

Damp/wet pre-lambing pens

Damp/wet nursery pens

Poor ventilation in lambing sheds

Hygiene of post- lambing pens

Damp/wet separate individual mothering pens

Muddy field conditions and/or standing water in fields

Early lambing (Feb- Mar)

Hygiene of nursery pens

Damp/wet post- lambing pens

Wet/poor weather

Middle lambing (Mar- Apr)

Late lambing (Apr- Jun)

Mixed indoor and outdoor lambing

Indoor lambing Outdoor lambing

Upland farms Lowland farms


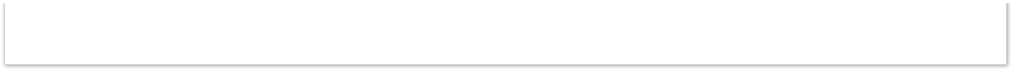


Other

If you selected other, please specify:


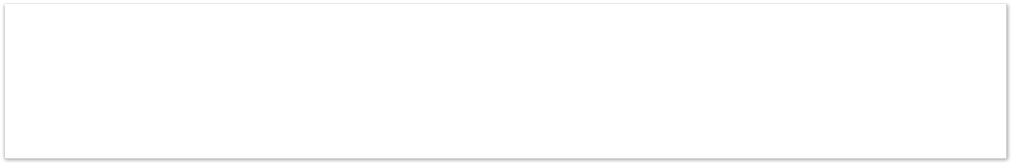


If a vaccine for joint ill was available, how likely would you be to use it?


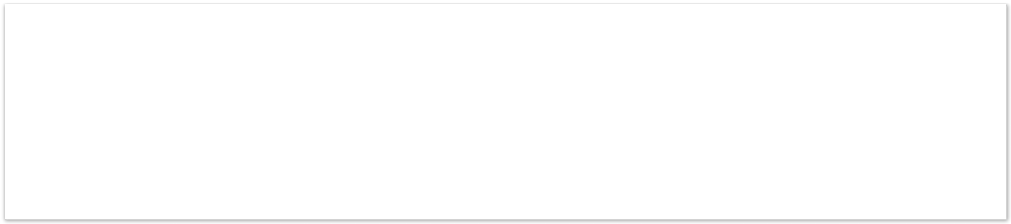

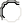

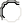

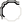

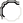


Very likely Likely Unlikely Not at all

1. The following questions are about the lambing environment.

Do you lamb this flock outdoors?


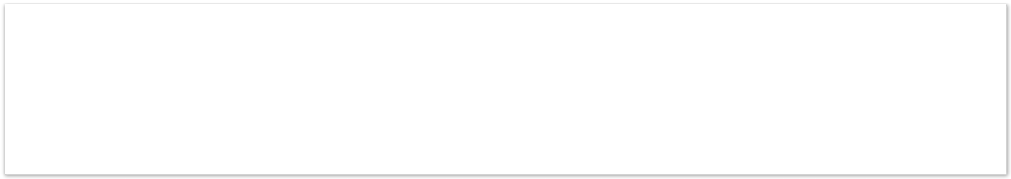

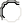

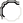

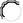


Yes No Mixed

D. The following questions are about the lambing environment.

For ewes that lamb outdoors, please estimate the stocking density (in ewes/hectare)


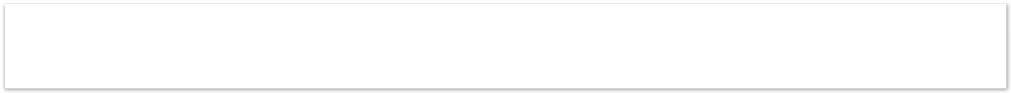


Do you provide additional shelter in lambing fields?


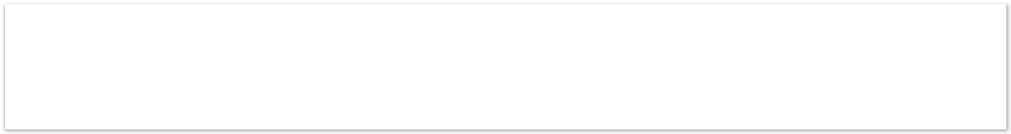

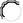

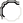


Yes No

How long after birth are ewes and lambs moved on?


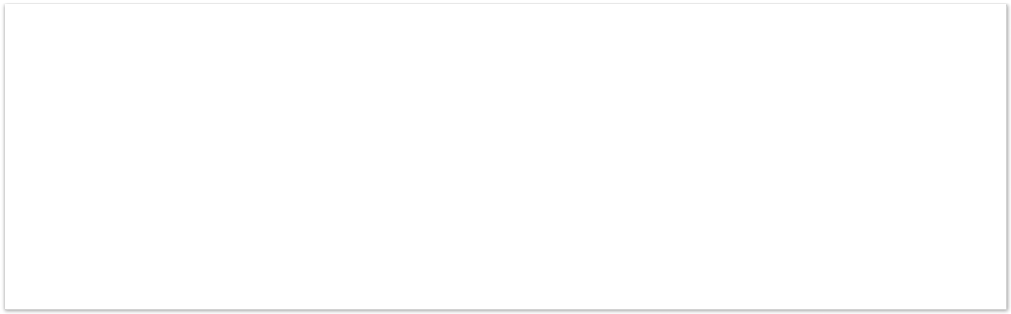

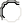

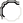

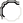

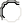

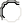

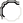


Within 24 hours

1-3 days

4-7 days Over a week

Set stock - not moved on Varied

If this varies, please explain why (e.g. individual animal circumstances/weather conditions):


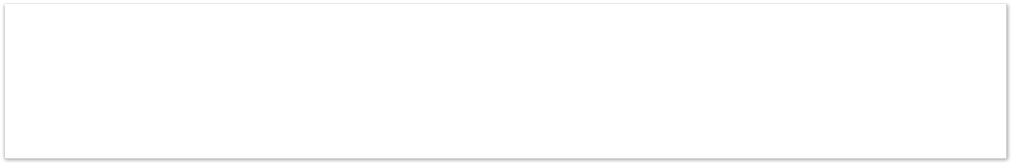


D. The following questions are about the lambing environment.

Do you lamb this flock indoors?


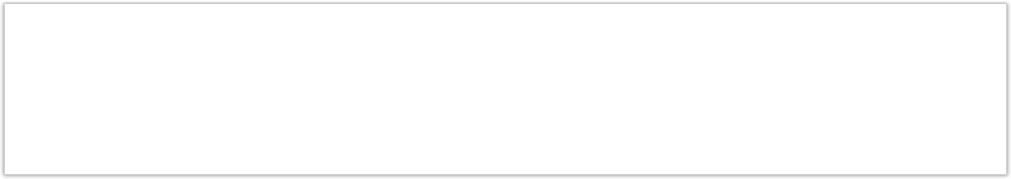

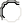

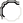

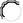


Yes No Mixed

D. The following questions are about the lambing environment.

How long are ewes housed before lambing? (weeks)


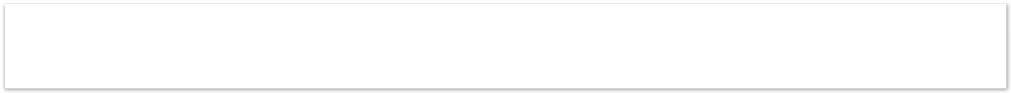


What is the stocking rate of ewes in group pens pre-lambing?

| Ewes per square feet |  |
| --- | --- |
| Ewes per square metre |  |

Do you clean out group pens before lambing starts?


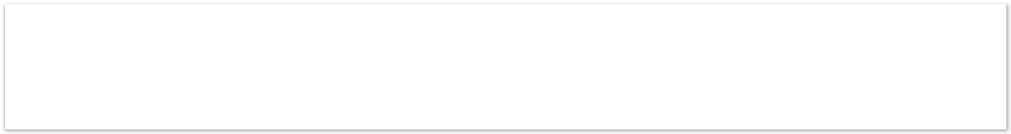


Yes No

Do you use separate individual mothering pens for ewes and lambs after birth?

Yes No

Sometimes

If sometimes, please describe the circumstances in which you use individual mothering pens?

How many separate individual mothering pens do you have available?

What type of bedding do you use in separate individual mothering pens?

Straw Shavings Paper

No bedding Other

If you selected other, please specify:

How often do you clean separate individual mothering pens? (Please tick all that apply)

Clean out bedding between every ewe

Top up of fresh bedding between every ewe

Clean pens when they are dirty/soiled/when it is required Clean pens approximately once a week

Clean pens at the end of the lambing season Other

If you selected other, please specify:

Do you regularly disinfect separate individual mothering pens when cleaning?

Yes No

If yes, what disinfectant do you use?

How long do ewes and lambs stay in separate individual mothering pens?

24 hours

1-3 days

4-7 days Over a week Varied

If this varies, please explain why (e.g. individual animal circumstances):

D. The following questions are about the lambing environment.

Do you use group nursey pens for ewes and lambs before turn out?

Yes No

Sometimes

If sometimes, please describe the circumstances in which you use group nursery pens? (e.g. only in poor weather)

D. The following questions are about the lambing environment.

What type of bedding do you use in group nursey pens?

Straw Shavings Paper

No bedding Other

If you selected other, please specify:

How often do you clean group nursey pens? (Please tick all that apply)

Clean pens when they are dirty/soiled/when it is required Clean pens every day

Clean pens every couple of days

Clean pens approximately once a week Clean pens between every group of ewes

Top up of fresh bedding between every group of ewes Clean pens at the end of the lambing season

Other

If you selected other, please specify:

Do you regularly disinfect group nursery pens when cleaning?

Yes No

If yes, what disinfectant do you use?

How long do ewes and lambs stay in group nursery pens?

24 hours

1-3 days

4-7 days Over a week Varied

If this varies, please explain why (e.g. individual animal circumstances):

D. The following questions are about the lambing environment.

In total, how long do ewes and lambs typically remain indoors before turnout?

24 hours

1-3 days

4-7 days Over a week Varies

If this varies, please explain why (e.g. singles let out sooner than doubles or triplets):

1. The following questions relate to the colostrum policy on your farm.

Do you body condition score ewes before lambing?

Yes No

If yes, what is the body condition score target for your flock?

Do you monitor ewes and lambs for colostrum intake?

Yes No

Sometimes

If yes, how do you do this? (Please tick all that apply)

Check lambs for fullness of stomach Check for colostrum in ewe udder Watch lambs suckling

Vet monitors via blood tests Other

If you selected other, please specify:

Do you administer supplemental colostrum?

Yes No

E. The following questions relate to the colostrum policy on your farm.

Which lambs do you administer colostrum to? (Please tick all that apply)

All lambs

Lambs who do not immediately suckle

Lambs who do not suckle within the first 6 hours of birth Lambs who do not suckled within the first 24 hours of birth All doubles

All triples

Those born to old/poor body condition ewes Other

If you selected other, please specify:

What is the source of the supplemental colostrum you administer? (Please tick all that apply)

Ewe's own colostrum

Fresh colostrum from another ewe Frozen colostrum from another ewe Artificial colostrum

Cow colostrum Other

If you selected Other, please specify:

Approximately, how much colostrum do you give to each lamb when supplementing? (in ml)

Do you regularly clean and/or disinfect stomach tubes?

Yes No

If yes, how often are they cleaned and/or disinfected? (Please tick all that apply)

Between each lamb Daily

Weekly

Only after dosing sick lambs Between each ewe

Do you regularly clean and/or disinfect lamb feeding equipment? (including bottles and teats)

Yes

No

If yes, how often are they cleaned and/or disinfected?

Between each lamb Daily

Weekly

Only after feeding sick lambs

1. The following questions relate to lamb management practices.

How many staff (including yourself) supervise the lambing flock?

Do you routinely 'dag'/'crutch' ewes before lambing?

Yes

No

Only if dirty

Do staff wear gloves when lambing ewes?

Yes

No

Sometimes

Do staff wash and/or disinfect hands between lambing ewes?

Yes

No

Sometimes

If yes, what do staff use to wash and/or disinfect hands? (Please tick all that apply)

Water

Soap and Water Alcohol gel

Disinfectant (e.g. hibiscrub) and water Other

If you selected other, please specify:

How often do you routinely clean and/or disinfect lambing ropes or head snares?

Between each use Daily

Weekly

1. The following question are about routine lambing timemanagement procedures

Do you treat lamb navels after lambing?

Yes No

How often do you treat lambs navels?

Once Twice

When do you treat lambs navels? (Please tick all that apply)

Within 2 hours of birth Within 6 hours of birth Within 12 hours of birth Other

If you selected other, please specify:

What product do you use to treat lambs navels?

Iodine Antibiotics

Other disinfectant

If you selected other disinfectant, please specify what you use:

How do you administer the treatment?

Spray Dip Other

If you selected other, please specify:

Do you ear tag lambs?

Yes No

At approximately what age do you ear tag lambs? (in days)

Are ear tags and equipment cleaned and/or disinfected before use?

Yes No

Do you castrate lambs?

Yes No

At what age are lambs typically castrated?

Within 6 hours of birth Within 24 hours of birth

Between 1 day and 1 week old Older than 1 week

Do clean and/or disinfect castration equipment before use?

Yes No

Do you tail dock lambs?

Yes No

At what age are lambs typically tail docked?

Within 6 hours of birth Within 24 hours of birth

Between 1 day and 1 week old Older than 1 week

Do you clean and/or disinfect tail docking equipment, including rubber rings, before use?

Yes No

For the chance to win an **Apple iPad**, a **Samsung Galaxy Tablet**, or a **Fortnum and Mason luxury hamper,** please enter your details at:

https://liverpool.onlinesurveys.ac.uk/prize-draw-for-joint-ill-survey

Your survey answers will **not** be linked to your entry details.

This project will be undertaking farm sampling and trial work. If you would be interested in taking part in further studies on the control of joint ill, please leave your contact details at:

https://liverpool.onlinesurveys.ac.uk/further-participation-in-joint-ill-study

and a member of the team will get back to you.

Your survey answers will **not** be linked to your contact details.

Contact Details

If you would like any more information on the survey or the project, please do not hesitate to contact Louise Jackson at:

[louise.jackson@liverpool.ac.uk](mailto:louise.jackson@liverpool.ac.uk)

Thank you for your participation!

Key for selection options

**1 - In which county is your farm?**

Aberdeenshire Angus

Antrim

Argyll & Bute Armagh Ayrshire Banffshire

Bath and North East Somerset Bedfordshire

Berkshire Berwickshire Blaenau Gwent Scottish Borders Bridgend

Conwy Cornwall County Durham Cumbria Denbighshire Derbyshire Devon

Dorset Down

Dumfries & Galloway Dunbartonshire

East Ayrshire

East Dunbartonshire East Lothian

East Renfrewshire

East Riding of Yorkshire East Sussex

Essex Fermanagh Fife Flintshire

Gloucestershire Greater London Greater Manchester Gwynedd Hampshire Herefordshire Hertfordshire Highland Inverclyde

Isle of Anglesey Isle of Wight Isles of Scilly Kent Kincardineshire Lanarkshire Lancashire Leicestershire Lincolnshire Londonderry

Merseyside Merthyr Tydfil Midlothian Monmouthshire Moray

Neath Port Talbot. Newport.

Norfolk

North Ayrshire North Lanarkshire North Somerset North Yorkshire Northamptonshire Northumberland Nottinghamshire Orkney Oxfordshire Pembrokeshire Perth & Kinross Powys

Renfrewshire

Rhondda Cynon Taff Rutland

Shetland Shropshire Somerset South Ayrshire

South Gloucestershire South Lanarkshire South Yorkshire Staffordshire Stirlingshire

Suffolk Surrey Swansea Torfaen

Tyne & Wear Tyrone

Vale of Glamorgan Warwickshire

West Dunbartonshire West Lothian

West Midlands West Sussex West Yorkshire Western Isles Wiltshire Worcester

hire Wrexham
