# Supplementary figures and images for: A cross-sectional survey of farmer reported prevalence and farm management practices associated with neonatal infectious arthritis (“joint ill”) in lambs, on UK sheep farms
Source: Front Vet Sci. 2024 Dec 23;11:1489751. doi: 10.3389/fvets.2024.1489751 (PMC11701153; doi:10.3389/fvets.2024.1489751)

**Supplementary Material 2: Causal diagram of NIA. Created BioRender.com.**


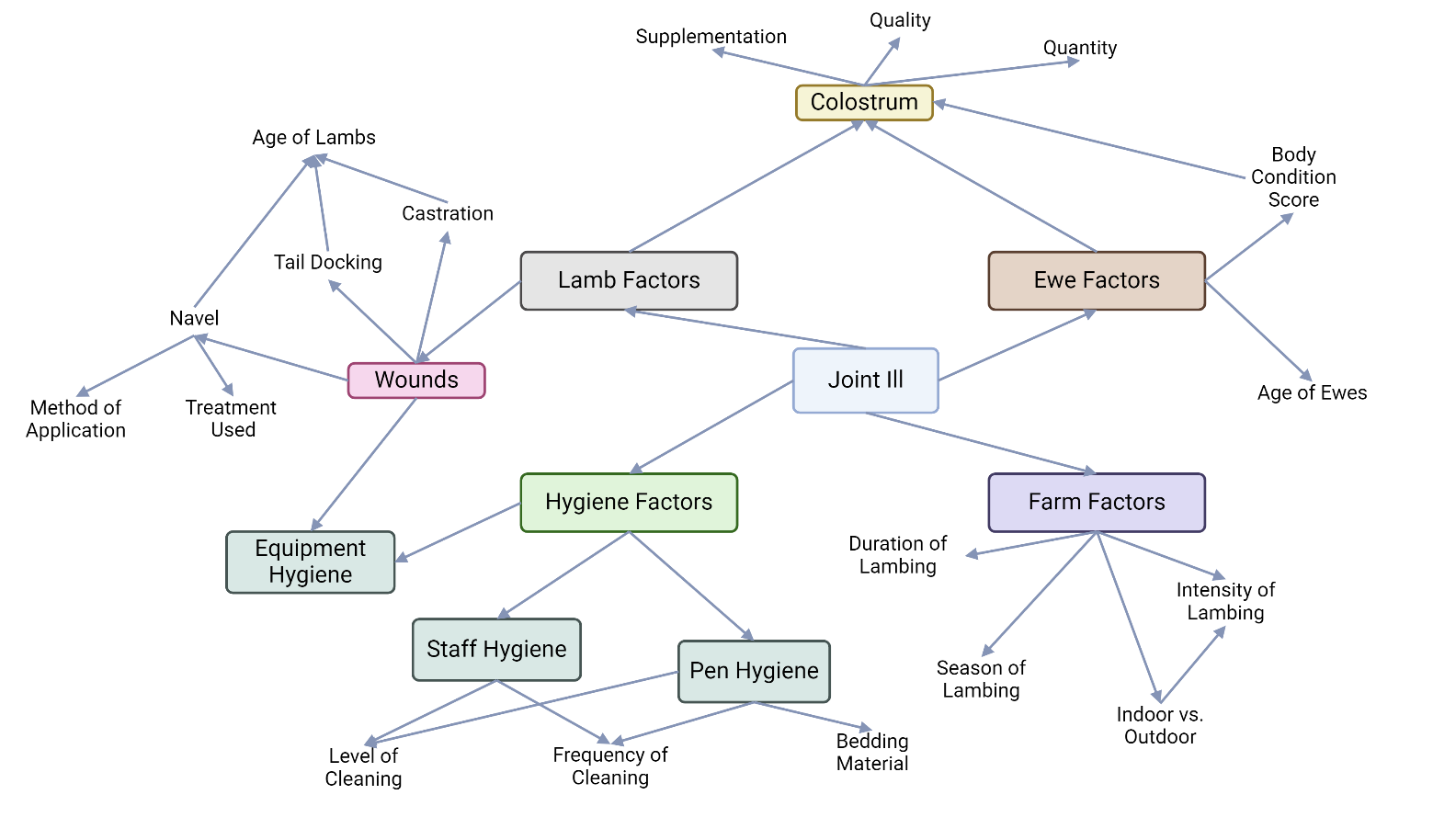

Supplement: Supplementary file 2 [file Table_2.docx]
