# Supplementary material for: A cross-sectional survey of farmer reported prevalence and farm management practices associated with neonatal infectious arthritis (“joint ill”) in lambs, on UK sheep farms
Source: Front Vet Sci. 2024 Dec 23;11:1489751. doi: 10.3389/fvets.2024.1489751 (PMC11701153; doi:10.3389/fvets.2024.1489751)
Supplement: Supplementary file 3 [file Table_3.DOCX]

**Supplementary Material 3: Full univariable analysis for the indoor lambing flocks dataset.**

| Category | Variable  (nr= number of responses) | Number of farms with Joint Ill | Percentage of farms with Joint Ill | Odds Ratio | 95% CI | P value |
| --- | --- | --- | --- | --- | --- | --- |
| Ewe Factors | **Age of Ewes (nr=232)** | **153** | **65.95%** |  |  | **0.834** |
|  | Majority over 5 years old (baseline) (nr=4) | 3 | 75.00% |  |  |  |
|  | 1-2 years (nr=2) | 1 | 50.00% | 0.33 | 0.01, 11.94 | 0.547 |
|  | Mixed ages (nr=226) | 149 | 65.93% | 0.65 | 0.07, 6.31 | 0.706 |
|  |  |  |  |  |  |  |
|  | **Body Condition Score Target (nr=116)** | **79** | **68.10%** |  |  | **0.227** |
|  | ≤3 (baseline) (nr=69) | 44 | 63.77% |  |  |  |
|  | >3 (nr=47) | 35 | 74.47% | 0.60 | 0.27, 1.37 | 0.227 |
|  |  |  |  |  |  |  |
|  | **‘Dagging’ Ewes (nr=233)** | **154** | **66.09%** |  |  | **0.434** |
|  | Yes (baseline) (nr=101) | 69 | 68.32% |  |  |  |
|  | No (nr=62) | 43 | 69.35% | 1.05 | 0.53, 2.079 | 0.890 |
|  | Only if dirty (nr=70) | 42 | 60.00% | 0.70 | 0.37, 1.31 | 0.263 |
|  |  |  |  |  |  |  |
|  | **Pure Bred vs. Cross Bred Ewes (nr=231)** | **152** | **65.80%** |  |  | **0.001** |
|  | Cross bred (baseline) (nr=155) | 113 | 72.90% |  |  |  |
|  | Pure bred (nr=76) | 39 | 51.32% | 0.39 | 0.22, 0.70 | 0.001 |
|  |  |  |  |  |  |  |
| Farm Factors | **Flock Type (nr=222)** | **150** | **67.57%** |  |  | **0.021** |
|  | Lowland (baseline) (nr=147) | 90 | 61.22% |  |  |  |
|  | Upland (nr=71) | 57 | 80.28% | 2.58 | 1.32, 5.05 | 0.006 |
|  | Mountain (nr=4) | 3 | 75.00% | 1.90 | 0.19, 18.71 | 0.582 |
|  |  |  |  |  |  |  |
|  | **Organic vs. Non-organic (nr=234)** | **155** | **66.24%** |  |  | **0.201** |
|  | Non-Organic (baseline) (nr=227) | 152 | 66.96% |  |  |  |
|  | Organic (nr=7) | 3 | 42.86% | 0.37 | 0.08, 1.70 | 0.201 |
|  |  |  |  |  |  |  |
|  | **Lambing Duration (weeks) (nr=230)** | **152** | **66.09%** | **1.10** | **0.97, 1.25** | **0.126** |
|  |  |  |  |  |  |  |
|  | **Lambing Period (nr=230)** | **151** | **65.65%** |  |  | **0.029** |
|  | Middle (baseline) (Feb-Mar) (nr=147) | 101 | 68.71% |  |  |  |
|  | Early (Sep-Jan) (nr=21) | 8 | 38.10% | 0.28 | 0.12, 0.72 | 0.008 |
|  | Late (Apr-May) (nr=62) | 42 | 67.74% | 0.96 | 0.51, 1.81 | 0.891 |
|  |  |  |  |  |  |  |
|  | **Scanning % (nr=179)** | **113** | **63.13%** |  |  | **0.486** |
|  | 151-200 (baseline) (nr=135) | 86 | 63.70% |  |  |  |
|  | < or equal to 150 (nr=28) | 19 | 67.86% | 1.20 | 0.51, 2.86 | 0.676 |
|  | 201+ (nr=16) | 8 | 50.00% | 0.57 | 0.20, 1.61 | 0.289 |
|  |  |  |  |  |  |  |
|  | **No. Ewes Lambed (nr=222)** | **147** | **66.22%** |  |  | **<0.001** |
|  | 1-100 (baseline) (nr=55) | 23 | 41.81% |  |  |  |
|  | 101-300 (nr=59) | 36 | 61.02% | 2.18 | 1.03, 4.61 | 0.042 |
|  | 301-600 (nr=55) | 43 | 78.18% | 4.99 | 2.16, 11.49 | <0.001 |
|  | 601+ (nr=53) | 45 | 84.91% | 7.83 | 3.12, 19.70 | <0.001 |
|  |  |  |  |  |  |  |
|  | **Lambs Born Alive (nr=219)** | **142** | **64.84%** |  |  | **<0.001** |
|  | 1-160 (baseline) (nr=54) | 22 | 40.74% |  |  |  |
|  | 161-500 (nr=60) | 36 | 60.00% | 2.18 | 1.03, 4.62 | 0.041 |
|  | 501-1000 (nr=53) | 39 | 73.58% | 4.05 | 1.79, 9.17 | 0.001 |
|  | 1001+ (nr=52) | 45 | 86.54% | 9.35 | 3.57, 24.51 | <0.001 |
|  |  |  |  |  |  |  |
|  | **Time Indoors Before Lambing (weeks) (nr=205)** | **136** | **66.34%** | **0.99** | **0.93, 1.06** | **0.793** |
|  |  |  |  |  |  |  |
|  | **Use of Mothering Pens (nr=234)** | **155** | **66.24%** |  |  | **n/a** |
|  | Yes (nr=221) | 146 | 66.06% |  |  |  |
|  | No (nr=1) | 1 | 100.00% |  |  |  |
|  | Sometimes (nr=12) | 8 | 66.67% |  |  |  |
|  |  |  |  |  |  |  |
|  | **Quantity of Mothering Pens (nr=211)** | **142** | **67.30%** | **1.01** | **1.00, 1.02** | **0.006** |
|  |  |  |  |  |  |  |
|  | **No. of Ewes Lambed per Quantity of Mothering Pens (nr=202)** | **135** | **66.83%** | **1.15** | **1.07, 1.24** | **<0.001** |
|  |  |  |  |  |  |  |
|  | **Bedding in Mothering Pens (nr=233)** | **154** | **66.09%** |  |  | **0.999** |
|  | Straw (baseline) (nr=218) | 144 | 66.06% |  |  |  |
|  | Shavings (nr=12) | 8 | 66.67% | 1.03 | 0.30, 3.53 | 0.965 |
|  | Other (nr=3) | 2 | 66.67% | 1.03 | 0.09, 11.52 | 0.982 |
|  |  |  |  |  |  |  |
|  | **Time Spent in Mothering Pens (nr=232)** | **153** | **65.95%** |  |  | **0.427** |
|  | < or equal to 24 hours (baseline) (nr=62) | 45 | 72.58% |  |  |  |
|  | >24 hours (nr=87) | 56 | 64.37% | 0.68 | 0.34,1.39 | 0.291 |
|  | Varied (nr=83) | 52 | 62.65% | 0.63 | 0.31, 1.29 | 0.210 |
|  |  |  |  |  |  |  |
|  | **Use of Nursery Pens (nr=233)** | **155** | **66.53%** |  |  | **0.200** |
|  | Yes (baseline) (nr=130) | 86 | 66.15% |  |  |  |
|  | No (nr=36) | 20 | 55.56% | 0.64 | 0.30, 1.36 | 0.243 |
|  | Sometimes (nr=67) | 49 | 73.13% | 1.39 | 0.73, 2.67 | 0.319 |
|  |  |  |  |  |  |  |
|  | **Bedding in Nursery Pens (nr=233)** | **154** | **66.09%** |  |  | **0.307** |
|  | Straw (baseline) (nr=195) | 133 | 68.21% |  |  |  |
|  | Shavings (nr=2) | 1 | 50.00% | 0.47 | 0.03, 7.58 | 0.592 |
|  | Does Not Use Nursery Pens (nr=36) | 20 | 55.56% | 0.58 | 0.28, 1.20 | 0.143 |
|  |  |  |  |  |  |  |
|  | **Time Spent in Nursery Pens (nr=234)** | **155** | **66.24%** |  |  | **0.129** |
|  | 24 hours (baseline) (nr=25) | 19 | 76.00% |  |  |  |
|  | 1-3 days (nr=88) | 65 | 73.86% | 0.89 | 0.32, 2.51 | 0.829 |
|  | >3 days (nr=29) | 19 | 65.52% | 0.60 | 0.18, 1.98 | 0.402 |
|  | Varied (nr=56) | 32 | 57.14% | 0.42 | 0.15, 1.22 | 0.110 |
|  | Does Not Use Nursery Pens (nr=36) | 20 | 55.56% | 0.40 | 0.13, 1.22 | 0.107 |
|  |  |  |  |  |  |  |
|  | **Total Time Indoors (nr=233)** | **155** | **66.52%** |  |  | **0.421** |
|  | 24 hours (baseline) (nr=23) | 15 | 65.22% |  |  |  |
|  | 1-3 days (nr=104) | 75 | 72.12% | 1.38 | 0.53, 3.60 | 0.511 |
|  | >3 days (nr=63) | 38 | 60.32% | 0.81 | 0.30, 2.19 | 0.679 |
|  | Varied (nr=43) | 27 | 62.79% | 0.90 | 0.31, 2.59 | 0.845 |
|  |  |  |  |  |  |  |
| Hygiene Factors | **Cleaning Group Pens (nr=231)** | **152** | **65.80%** |  |  | **0.783** |
|  | Yes (baseline) (nr=167) | 109 | 65.27% |  |  |  |
|  | No (nr=64) | 43 | 67.19% | 1.09 | 0.59, 2.01 | 0.783 |
|  |  |  |  |  |  |  |
|  | **Cleaning of Mothering Pens (nr=231)** | **153** | **66.23%** |  |  | **0.051** |
|  | Between every ewe (baseline) (nr=109) | 62 | 56.88% |  |  |  |
|  | When dirty/soiled/required (nr=35) | 24 | 68.57% | 1.65 | 0.74, 3.71 | 0.222 |
|  | Top up of fresh bedding between ewes (nr=82) | 64 | 78.05% | 2.70 | 1.41, 5.14 | 0.003 |
|  | Infrequently (less than once a week) (nr=3) | 2 | 66.67% | 1.51 | 0.13, 17.23 | 0.737 |
|  | Other (nr=2) | 1 | 50.00% | 0.76 | 0.05, 12.44 | 0.846 |
|  |  |  |  |  |  |  |
|  | **Disinfecting Mothering Pens (nr=231)** | **154** | **66.67%** |  |  | **0.418** |
|  | Yes (baseline) (nr=161) | 110 | 68.32% |  |  |  |
|  | No (nr=70) | 44 | 62.86% | 0.79 | 0.44, 1.41 | 0.418 |
|  |  |  |  |  |  |  |
|  | **Cleaning of Nursery Pens (nr=227)** | **152** | **66.96%** |  |  | **0.258** |
|  | Between every group of ewes (baseline) (nr=14) | 7 | 50.00% |  |  |  |
|  | When dirty/soiled/required (nr=39) | 27 | 69.23% | 2.25 | 0.65, 7.85 | 0.203 |
|  | Every couple of days (nr=5) | 2 | 40.00% | 0.67 | 0.08, 5.30 | 0.702 |
|  | Every day (nr=8) | 7 | 87.50% | 7.00 | 0.67, 72.86 | 0.104 |
|  | Top up fresh bedding between every group of ewes (nr=101) | 72 | 71.29% | 2.48 | 0.80, 7.71 | 0.116 |
|  | Infrequently (less than once a week) (nr=24) | 17 | 79.83% | 2.43 | 0.62, 9.54 | 0.204 |
|  | Does Not Use Nursery Pens (nr=36) | 20 | 55.56% | 1.25 | 0.36, 4.31 | 0.724 |
|  |  |  |  |  |  |  |
|  | **Nursery Pens Disinfected (nr=231)** | **152** | **65.80%** |  |  | **0.353** |
|  | Yes (baseline) (nr=77) | 51 | 66.23% |  |  |  |
|  | No (nr=118) | 81 | 68.64% | 1.12 | 0.61, 2.06 | 0.725 |
|  | Does Not Use Nursery Pens (nr=36) | 20 | 55.56% | 0.64 | 0.28, 1.43 | 0.275 |
|  |  |  |  |  |  |  |
|  | **Cleaning Stomach Tubes (nr=233)** | **154** | **66.09%** |  |  | **0.202** |
|  | Yes (baseline) (nr=181) | 125 | 69.06% |  |  |  |
|  | No (nr=4) | 2 | 50.00% | 0.45 | 0.06, 3.26 | 0.428 |
|  | Does Not Supplement (nr=48) | 27 | 56.25% | 0.5760 | 0.30, 1.12 | 0.097 |
|  |  |  |  |  |  |  |
|  | **Freq. of Cleaning Stomach Tubes (nr=233)** | **154** | **66.09%** |  |  | **0.034** |
|  | Between each lamb and/or ewe (baseline) (nr=150) | 96 | 64.00% |  |  |  |
|  | Daily (nr=27) | 25 | 92.59% | 7.03 | 1.60, 30.84 | 0.010 |
|  | Does Not or Infrequently Cleans Stomach Tubes (nr=8) | 6 | 75.00% | 1.69 | 0.33, 8.65 | 0.530 |
|  | Does Not Supplement (nr=48) | 27 | 56.25% | 0.72 | 0.37, 1.40 | 0.336 |
|  |  |  |  |  |  |  |
|  | **Cleaning Bottles (nr=232)** | **153** | **65.95%** |  |  | **n/a** |
|  | Yes (nr=182) | 124 | 68.13% |  |  |  |
|  | No (nr=2) | 2 | 100.00% |  |  |  |
|  | Does Not Supplement (nr=48) | 27 | 56.25% |  |  |  |
|  |  |  |  |  |  |  |
|  | **Freq. of Cleaning Bottles (nr=231)** | **152** | **65.80%** |  |  | **0.047** |
|  | Between each lamb (baseline) (nr=112) | 69 | 61.61% |  |  |  |
|  | Daily (nr=66) | 52 | 78.79% | 2.32 | 1.15, 4.67 | 0.019 |
|  | Does Not or Infrequently Cleans Bottles (nr=5) | 4 | 80.00% | 2.49 | 0.27, 23.05 | 0.421 |
|  | Does Not Supplement (nr=48) | 27 | 56.25% | 0.80 | 0.40, 1.59 | 0.526 |
|  |  |  |  |  |  |  |
|  | **Freq. of Cleaning Lambing Ropes and Head Snares (nr=223)** | **148** | **66.37%** |  |  | **0.647** |
|  | Between Each Use (baseline) (nr=193) | 126 | 65.28% |  |  |  |
|  | Daily (nr=17) | 12 | 70.59% | 1.28 | 0.43, 3.78 | 0.659 |
|  | Weekly (nr=13) | 10 | 76.92% | 1.77 | 0.47, 6.66 | 0.397 |
|  |  |  |  |  |  |  |
|  | **Wearing Gloves (nr=234)** | **155** | **66.24%** |  |  | **0.689** |
|  | Yes (baseline) (nr=102) | 69 | 67.65% |  |  |  |
|  | No (nr=132) | 86 | 65.15% | 0.89 | 0.52, 1.55 | 0.689 |
|  |  |  |  |  |  |  |
|  | **Washing Hands (nr=234)** | **155** | **66.24%** |  |  | **0.002** |
|  | Yes (baseline) (nr=178) | 108 | 60.67% |  |  |  |
|  | No or Sometimes (nr=56) | 47 | 83.93% | 0.30 | 0.14, 0.64 | 0.002 |
|  |  |  |  |  |  |  |
|  |  |  |  |  |  |  |
|  | **Method of Hand Washing (nr=230)** | **151** | **65.65%** |  |  | **0.344** |
|  | Disinfectant (baseline) (nr=33) | 22 | 66.67% |  |  |  |
|  | Alcohol gel (nr=4) | 2 | 50.00% | 0.50 | 0.06, 4.04 | 0.516 |
|  | Soap and Water (nr=130) | 79 | 60.77% | 0.78 | 0.35, 1.73 | 0.534 |
|  | Water (nr=8) | 6 | 75.00% | 1.50 | 0.26, 8.69 | 0.651 |
|  | Combination of methods (nr=43) | 31 | 72.09% | 1.29 | 0.48, 3.46 | 0.610 |
|  | Does Not Wash Hands (nr=12) | 11 | 91.67% | 5.50 | 0.63, 48.19 | 0.124 |
|  |  |  |  |  |  |  |
| Lamb Factors | **Using Preventative Measures for JI (nr=233)** | **155** | **66.52%** |  |  | **0.014** |
|  | Yes (baseline) (nr=170) | 121 | 71.18% |  |  |  |
|  | No (nr=63) | 34 | 53.97% | 0.48 | 0.26, 0.86 | 0.014 |
|  |  |  |  |  |  |  |
|  | **Antibiotics as Preventative Measure (nr=232)** | **154** | **66.38%** |  |  | **0.013** |
|  | Yes (baseline) (nr=28) | 16 | 57.14% |  |  |  |
|  | No (nr=141) | 104 | 73.76% | 2.11 | 0.91, 4.87 | 0.081 |
|  | Does Not Use Preventative Measures (nr=63) | 34 | 53.97% | 0.88 | 0.36, 2.16 | 0.779 |
|  |  |  |  |  |  |  |
|  | **Lambs Given Antibiotics for Prevention (nr=232)** | **154** | **66.38%** |  |  | **0.061** |
|  | All lambs born after JI cases occur (baseline) (nr=8) | 7 | 87.50% |  |  |  |
|  | Only specific high-risk groups (nr=20) | 11 | 55.00% | 0.18 | 0.02, 1.70 | 0.132 |
|  | All lambs whether or not JI present (nr=17) | 12 | 70.59% | 0.34 | 0.03, 3.56 | 0.370 |
|  | Does Not Use Antibiotics (nr=124) | 90 | 72.58% | 0.38 | 0.05, 3.19 | 0.371 |
|  | Does Not Use Preventative Measures (nr=63) | 34 | 53.97% | 0.17 | 0.02, 1.44 | 0.104 |
|  |  |  |  |  |  |  |
|  | **Monitoring Colostrum (nr=232)** | **153** | **65.95%** |  |  | **0.727** |
|  | Yes (baseline) (nr=214) | 142 | 66.36% |  |  |  |
|  | No (nr=4) | 3 | 75.00% | 1.52 | 0.16, 14.88 | 0.719 |
|  | Sometimes (nr=14) | 8 | 57.14% | 0.68 | 0.23, 2.02 | 0.484 |
|  |  |  |  |  |  |  |
|  | **Supplementing Colostrum (nr=234)** | **155** | **66.24%** |  |  | **0.103** |
|  | Yes (baseline) (nr=186) | 128 | 68.82% |  |  |  |
|  | No (nr=48) | 27 | 56.25% | 0.58 | 0.30, 1.12 | 0.103 |
|  |  |  |  |  |  |  |
|  | **Treating Navels (nr=232)** | **154** | **66.38%** |  |  | **0.390** |
|  | Yes (baseline) (nr=226) | 149 | 65.93% |  |  |  |
|  | No (nr=6) | 5 | 83.33% | 2.58 | 0.30, 22.51 | 0.390 |
|  |  |  |  |  |  |  |
|  | **Freq. of Navel Treatment (nr=230)** | **152** | **66.09%** |  |  | **0.524** |
|  | Once (baseline) (nr=150) | 96 | 64.00% |  |  |  |
|  | Twice (nr=74) | 51 | 68.92% | 1.25 | 0.69, 2.26 | 0.466 |
|  | Does Not Treat Navels (nr=6) | 5 | 83.33% | 2.81 | 0.32, 24.70 | 0.351 |
|  |  |  |  |  |  |  |
|  | **Navel Treatment Used (nr=234)** | **155** | **66.24%** |  |  | **0.783** |
|  | Iodine (baseline) (nr=205) | 136 | 66.34% |  |  |  |
|  | Antibiotics (nr=3) | 2 | 66.67% | 1.02 | 0.09, 11.39 | 0.991 |
|  | Other disinfectant (nr=20) | 12 | 60.00% | 0.76 | 0.30, 1.95 | 0.569 |
|  | Does Not Treat Navels (nr=6) | 5 | 83.33% | 2.54 | 0.29, 22.14 | 0.400 |
|  |  |  |  |  |  |  |
|  | **Navel Treatment Application (nr=234)** | **155** | **66.24%** |  |  | **0.669** |
|  | Spray (baseline) (nr=130) | 83 | 63.85% |  |  |  |
|  | Dip (nr=93) | 63 | 67.74% | 1.19 | 0.68, 2.09 | 0.546 |
|  | Other (nr=5) | 4 | 80.00% | 2.27 | 0.25, 20.86 | 0.470 |
|  | Does Not Treat Navels (nr=6) | 5 | 83.33% | 2.83 | 0.32, 24.96 | 0.349 |
|  |  |  |  |  |  |  |
|  | **Age of Navel Treatment (nr=234)** | **155** | **66.24%** |  |  | **0.739** |
|  | Within 2 hours of birth (immediately) (baseline) (nr=214) | 141 | 65.89% |  |  |  |
|  | Between 2 and 12 hours of birth (nr=10) | 7 | 70.00% | 1.21 | 0.30, 4.81 | 0.789 |
|  | Other (nr=4) | 2 | 50.00% | 0.52 | 0.07, 3.75 | 0.515 |
|  | Does Not Treat Navels (nr=6) | 5 | 83.33% | 2.59 | 0.30, 22.57 | 0.389 |
|  |  |  |  |  |  |  |
|  | **Ear Tagging (nr=232)** | **153** | **65.95%** |  |  | **0.066** |
|  | No (baseline) (nr=131) | 93 | 70.99% |  |  |  |
|  | Yes (nr=101) | 60 | 59.41% | 0.60 | 0.35, 1.03 | **0.066** |
|  |  |  |  |  |  |  |
|  | **Cleaning Ear Tags (nr=234)** | **155** | **66.24%** |  |  | **0.090** |
|  | Yes (baseline) (nr=52) | 28 | 53.85% |  |  |  |
|  | No (nr=51) | 34 | 66.67% | 1.71 | 0.77, 3.81 | 0.185 |
|  | Does Not Ear Tag (nr=131) | 93 | 70.99% | 2.10 | 1.08, 4.07 | 0.029 |
|  |  |  |  |  |  |  |
|  | **Age of Ear Tagging (nr=97)** | **58** | **59.79%** | **1.00** | **0.99, 1.02** | **0.805** |
|  |  |  |  |  |  |  |
|  | **Castration (nr=230)** | **151** | **65.65%** |  |  | **0.822** |
|  | No (baseline) (nr=75) | 50 | 66.67% |  |  |  |
|  | Yes (nr=155) | 101 | 65.16% | 0.94 | 0.52, 1.68 | 0.822 |
|  |  |  |  |  |  |  |
|  | **Cleaning Castration Equipment (nr=233)** | **154** | **66.09%** |  |  | **0.438** |
|  | Yes (baseline) (nr=51) | 30 | 58.82% |  |  |  |
|  | No (nr=107) | 74 | 69.16% | 1.57 | 0.79, 3.14 | 0.202 |
|  | Does Not Castrate (nr=75) | 50 | 66.67% | 1.40 | 0.67, 2.92 | 0.370 |
|  |  |  |  |  |  |  |
|  | **Age of Castration (nr=233)** | **154** | **66.09%** |  |  | **0.113** |
|  | Within 24 hours of birth (baseline) (nr=61) | 47 | 8.48% |  |  |  |
|  | Between 1 day and 1 week old (nr=93) | 54 | 58.06% | 0.41 | 0.20, 0.85 | 0.017 |
|  | Older than 1 week (nr=4) | 3 | 75.00% | 0.89 | 0.09, 9.28 | 0.925 |
|  | Does Not Castrate (nr=75) | 50 | 66.67% | 0.60 | 0.30, 1.28 | 0.185 |
|  |  |  |  |  |  |  |
|  | **Tail Docking (nr=231)** | **152** | **65.80%** |  |  | **0.170** |
|  | No (baseline) (nr=31) | 17 | 54.84% |  |  |  |
|  | Yes (nr=200) | 135 | 67.50% | 1.71 | 0.80, 3.68 | **0.170** |
|  |  |  |  |  |  |  |
|  | **Cleaning Tail Docking Equipment (nr=234)** | **155** | **66.24%** |  |  | **0.216** |
|  | Yes (baseline) (nr=59) | 37 | 62.71% |  |  |  |
|  | No (nr=144) | 101 | 70.14% | 1.40 | 0.74, 2.64 | 0.304 |
|  | Does Not Tail Dock (nr=31) | 17 | 54.84% | 0.72 | 0.30, 1.75 | 0.469 |
|  |  |  |  |  |  |  |
|  | **Age of Tail Docking (nr=233)** | **154** | **66.09%** |  |  | **0.053** |
|  | Within 24 hours of birth (baseline) (nr=79) | 61 | 77.22% |  |  |  |
|  | Between 1 day and 1 week old (nr=118) | 72 | 61.02% | 0.46 | 0.24, 0.88 | 0.019 |
|  | Older than 1 week (nr=5) | 4 | 80.00% | 1.18 | 0.12, 11.24 | 0.885 |
|  | Does Not Tail Dock (nr=31) | 17 | 54.84% | 0.36 | 0.15, 0.87 | 0.022 |
