# Supplementary material for: A cross-sectional survey of farmer reported prevalence and farm management practices associated with neonatal infectious arthritis (“joint ill”) in lambs, on UK sheep farms
Source: Front Vet Sci. 2024 Dec 23;11:1489751. doi: 10.3389/fvets.2024.1489751 (PMC11701153; doi:10.3389/fvets.2024.1489751)
Supplement: Supplementary file 4 [file Table_4.DOCX]

**Supplementary Material 4: Full univariable analysis for the outdoor lambing flocks dataset.**

| Category | Variable  (nr= number of responses) | Number of farms with Joint Ill | Percentage of farms with Joint Ill | Odds Ratio | 95% CI | P value |
| --- | --- | --- | --- | --- | --- | --- |
| Ewe Factors | **Age of Ewes (nr=161)** | **104** | **64.60%** |  |  | **0.155** |
|  | Majority over 5 years old (baseline) (nr=5) | 2 | 40.00% |  |  |  |
|  | 1-2 years (nr=6) | 2 | 33.33% | 0.75 | 0.06, 8.83 | 0.819 |
|  | Mixed ages (nr=150) | 100 | 66.67% | 3.00 | 0.49, 18.54 | 0.237 |
|  |  |  |  |  |  |  |
|  | **Body Condition Score Target (nr=94)** | **58** | **61.70%** |  |  | **0.375** |
|  | ≤3 (baseline) (nr=55) | 36 | 65.45% |  |  |  |
|  | >3 (nr=39) | 22 | 56.41% | 0.68 | 0.29, 1.59 | 0.375 |
|  |  |  |  |  |  |  |
|  | **‘Dagging’ Ewes (nr=163)** | **106** | **65.03%** |  |  | **0.726** |
|  | Yes (baseline) (nr=64) | 44 | 68.75% |  |  |  |
|  | No (nr=51) | 32 | 62.75% | 0.77 | 0.35, 1.66 | 0.500 |
|  | Only if dirty (nr=48) | 30 | 62.50% | 0.76 | 0.35, 1.67 | 0.490 |
|  |  |  |  |  |  |  |
|  | **Pure Bred vs. Cross Bred Ewes (nr=159)** | **103** | **64.78%** |  |  | **0.013** |
|  | Cross bred (baseline) (nr=81) | 60 | 74.07% |  |  |  |
|  | Pure bred (nr=78) | 43 | 55.13% | 0.43 | 0.22, 0.84 | 0.013 |
|  |  |  |  |  |  |  |
| Farm Factors | **Flock Type (nr=152)** | **99** | **65.13%** |  |  | **0.453** |
|  | Lowland (baseline) (nr=87) | 53 | 60.92% |  |  |  |
|  | Upland (nr=55) | 39 | 70.90% | 1.56 | 0.76, 3.23 | 0.226 |
|  | Mountain (nr=10) | 7 | 70.00% | 1.50 | 0.36, 6.19 | 0.578 |
|  |  |  |  |  |  |  |
|  | **Organic vs. Non-organic (nr=164)** | **107** | **65.24%** |  |  | **0.753** |
|  | Non-Organic (baseline) (nr=151) | 98 | 64.90% |  |  |  |
|  | Organic (nr=13) | 9 | 69.23% | 1.22 | 0.36, 4.14 | 0.753 |
|  |  |  |  |  |  |  |
|  | **Lambing Duration (weeks) (nr=159)** | **103** | **64.78%** | **1.23** | **1.03, 1.48** | **0.023** |
|  |  |  |  |  |  |  |
|  | **Lambing Period (nr=160)** | **103** | **64.38%** |  |  | **0.448** |
|  | Middle (baseline) (Feb-Mar) (nr=69) | 48 | 69.57% |  |  |  |
|  | Early (Sep-Jan) (nr=4) | 2 | 50.00% | 0.44 | 0.06, 3.32 | 0.424 |
|  | Late (Apr-May) (nr=87) | 53 | 60.92% | 0.68 | 0.35, 1.33 | 0.263 |
|  |  |  |  |  |  |  |
|  | **Scanning % (nr=125)** | **81** | **64.80%** |  |  | **0.316** |
|  | 151-200 (baseline) (nr=95) | 63 | 66.32% |  |  |  |
|  | < or equal to 150 (nr=18) | 9 | 50.00% | 0.51 | 0.18, 1.41 | 0.192 |
|  | 201+ (nr=12) | 9 | 75.00% | 1.52 | 0.39, 6.02 | 0.548 |
|  |  |  |  |  |  |  |
|  | **No. Ewes Lambed (nr=154)** | **100** | **64.94%** |  |  | **<0.001** |
|  | 1-100 (baseline) (nr=39) | 13 | 33.33% |  |  |  |
|  | 101-300 (nr=29) | 18 | 62.07% | 3.27 | 1.20, 8.92 | 0.021 |
|  | 301-600 (nr=42) | 29 | 69.05% | 4.46 | 1.75, 11.35 | 0.002 |
|  | 601+ (nr=44) | 40 | 90.90% | 20.00 | 5.88, 68.04 | <0.001 |
|  |  |  |  |  |  |  |
|  | **Lambs Born Alive (nr=153)** | **97** | **63.40%** |  |  | **<0.001** |
|  | 1-160 (baseline) (nr=38) | 12 | 31.58% |  |  |  |
|  | 161-500 (nr=29) | 17 | 58.62% | 3.07 | 1.12, 8.40 | 0.029 |
|  | 501-1000 (nr=46) | 33 | 71.74% | 5.50 | 2.15, 14.05 | <0.001 |
|  | 1001+ (nr=40) | 35 | 87.50% | 15.17 | 4.75, 48.39 | <0.001 |
|  |  |  |  |  |  |  |
|  | **Outdoor Shelter (nr=160)** | **104** | **65.00%** |  |  | **<0.001** |
|  | Yes (baseline) (nr=44) | 16 | 36.36% |  |  |  |
|  | No (nr=116) | 88 | 75.86% | 5.50 | 2.61, 11.61 | <0.001 |
|  |  |  |  |  |  |  |
|  | **Outdoor Moving on After Birth (nr=159)** | **102** | **64.15%** |  |  | **0.823** |
|  | Within 24 hours (baseline) (nr=31) | 21 | 67.74% |  |  |  |
|  | 1-3 days (nr=43) | 25 | 58.14% | 0.66 | 0.25, 1.74 | 0.402 |
|  | Greater than or equal to 4 days (nr=28) | 20 | 71.43% | 1.19 | 0.39, 3.62 | 0.759 |
|  | Set Stock – Not Moved on (nr=25) | 16 | 64.00% | 0.85 | 0.28, 2.57 | 0.769 |
|  | Varied (nr=32) | 20 | 62.50% | 0.79 | 0.28, 2.24 | 0.663 |
|  |  |  |  |  |  |  |
| Hygiene Factors | **Cleaning Stomach Tubes (nr=162)** | **105** | **64.81%** |  |  | **0.186** |
|  | Yes (baseline) (nr=102) | 71 | 69.61% |  |  |  |
|  | No (nr=4) | 3 | 75.00% | 1.31 | 0.13, 13.09 | 0.818 |
|  | Does Not Supplement (nr=56) | 31 | 55.36% | 0.54 | 0.28, 1.06 | 0.075 |
|  |  |  |  |  |  |  |
|  | **Freq. of Cleaning Stomach Tubes (nr=162)** | **105** | **64.81%** |  |  | **0.052** |
|  | Between each lamb and/or ewe (baseline) (nr=79) | 50 | 63.29% |  |  |  |
|  | Daily (nr=21) | 19 | 90.48% | 5.51 | 1.20, 25.37 | 0.028 |
|  | Does Not or Infrequently Cleans Stomach Tubes (less than daily) (nr=6) | 5 | 83.33% | 2.90 | 0.32, 26.05 | 0.342 |
|  | Does Not Supplement (nr=56) | 31 | 55.36% | 0.72 | 0.36, 1.45 | 0.355 |
|  |  |  |  |  |  |  |
|  | **Cleaning Bottles (nr=162)** | **105** | **64.81%** |  |  | **n/a** |
|  | Yes (nr=105) | 73 | 69.52% |  |  |  |
|  | No (nr=1) | 1 | 100.00% |  |  |  |
|  | Does Not Supplement (nr=56) | 31 | 55.36% |  |  |  |
|  |  |  |  |  |  |  |
|  | **Freq. of Cleaning Bottles (nr=162)** | **105** | **64.81%** |  |  | **n/a** |
|  | Between each lamb (baseline) (nr=63) | 40 | 63.49% |  |  |  |
|  | Daily (nr=39) | 31 | 79.49% |  |  |  |
|  | Does Not or Infrequently Cleans Bottles (less than daily) (nr=4) | 3 | 75.00% |  |  |  |
|  | Does Not Supplement (nr=56) | 31 | 55.36% |  |  |  |
|  |  |  |  |  |  |  |
|  | **Freq. of Cleaning Lambing Ropes and Head Snares (nr=156)** | **99** | **63.46%** |  |  | 0.704 |
|  | Between Each Use (baseline) (nr=126) | 78 | 61.90% |  |  |  |
|  | Daily (nr=16) | 11 | 68.75% | 1.35 | 0.44, 4.14 | 0.595 |
|  | Weekly (nr=14) | 10 | 71.43% | 1.54 | 0.46, 5.18 | 0.487 |
|  |  |  |  |  |  |  |
|  | **Wearing Gloves (nr=164)** | **107** | **65.24%** |  |  | **0.607** |
|  | Yes (baseline) (nr=59) | 40 | 67.80% |  |  |  |
|  | No (nr=105) | 67 | 63.81% | 0.84 | 0.43, 1.65 | 0.607 |
|  |  |  |  |  |  |  |
|  | **Washing Hands (nr=164)** | **107** | **65.24%** |  |  | **0.277** |
|  | Yes (baseline) (nr=114) | 70 | 61.40% |  |  |  |
|  | No (nr=10) | 8 | 80.00% | 2.51 | 0.51, 12.39 | 0.257 |
|  | Sometimes (nr=40) | 29 | 72.50% | 1.66 | 0.75, 3.65 | 0.210 |
|  |  |  |  |  |  |  |
|  | **Method of Hand Washing (nr=156)** | **102** | **65.38%** |  |  | **0.939** |
|  | Disinfectant (baseline) (nr=19) | 11 | 57.89% |  |  |  |
|  | Alcohol gel (nr=5) | 3 | 60.00% | 1.09 | 0.15, 8.12 | 0.932 |
|  | Soap and Water (nr=87) | 58 | 66.67% | 1.46 | 0.53, 4.01 | 0.469 |
|  | Just water (nr=5) | 3 | 60.00% | 1.09 | 0.15, 8.12 | 0.932 |
|  | Combination of methods (nr=31) | 20 | 64.52% | 1.32 | 0.41, 4.26 | 0.640 |
|  | Does Not Wash Hands (nr=9) | 7 | 77.78% | 2.55 | 0.41, 15.65 | 0.313 |
|  |  |  |  |  |  |  |
| Lamb Factors | **Using Preventative Measures for JI (nr=163)** | **107** | **65.64%** |  |  | **0.014** |
|  | Yes (baseline) (nr=100) | 73 | 73.00% |  |  |  |
|  | No (nr=63) | 34 | 53.97% | 0.43 | 0.22, 0.84 | 0.014 |
|  |  |  |  |  |  |  |
|  | **Antibiotics as Preventative Method (nr=163)** | **107** | **65.64%** |  |  | **0.020** |
|  | Yes (baseline) (nr=20) | 12 | 60.00% |  |  |  |
|  | No (nr=80) | 61 | 76.25% | 2.14 | 0.76, 6.01 | 0.148 |
|  | Does Not Use Preventative Measures (nr=63) | 34 | 53.97% | 0.78 | 0.28, 2.17 | 0.637 |
|  |  |  |  |  |  |  |
|  | **Lambs Given Antibiotics for Prevention (nr=162)** | **107** | **66.05%** |  |  | **n/a** |
|  | All lambs born after JI cases occur (baseline) (nr=4) | 4 | 100.00% |  |  |  |
|  | Only specific high-risk groups (nr=11) | 5 | 45.45% |  |  |  |
|  | All lambs whether or not JI present (nr=10) | 7 | 70.00% |  |  |  |
|  | Does Not Use Antibiotics (nr=75) | 57 | 76.00% |  |  |  |
|  | Does Not Use Preventative Measures (nr=62) | 34 | 54.84% |  |  |  |
|  |  |  |  |  |  |  |
|  | **Monitoring Colostrum (nr=163)** | **106** | **65.03%** |  |  | **0.683** |
|  | Yes (baseline) (nr=126) | 84 | 66.67% |  |  |  |
|  | No (nr=16) | 10 | 62.50% | 0.83 | 0.28, 2.45 | 0.740 |
|  | Sometimes (nr=21) | 12 | 57.14% | 0.67 | 0.26, 1.71 | 0.398 |
|  |  |  |  |  |  |  |
|  | **Supplementing Colostrum (nr=164)** | **107** | **65.24%** |  |  | **0.057** |
|  | Yes (baseline) (nr=108) | 76 | 70.37% |  |  |  |
|  | No (nr=56) | 31 | 55.36% | 0.52 | 0.27, 1.02 | 0.057 |
|  |  |  |  |  |  |  |
|  | **Treating Navels (nr=162)** | **105** | **64.81%** |  |  | **0.874** |
|  | Yes (baseline) (nr=129) | 84 | 65.12% |  |  |  |
|  | No (nr=33) | 21 | 63.63% | 0.94 | 0.42, 2.08 | 0.874 |
|  |  |  |  |  |  |  |
|  | **Freq. of Navel Treatment (nr=157)** | **103** | **65.01%** |  |  | **0.916** |
|  | Once (baseline) (nr=85) | 57 | 67.06% |  |  |  |
|  | Twice (nr=39) | 25 | 64.10% | 0.88 | 0.40, 1.94 | 0.747 |
|  | Does Not Treat Navels (nr=33) | 21 | 63.63% | 0.86 | 0.37, 1.99 | 0.725 |
|  |  |  |  |  |  |  |
|  | **Navel Treatment Used (nr=163)** | **106** | **65.03%** |  |  | **0.944** |
|  | Iodine (baseline) (nr=119) | 77 | 64.71% |  |  |  |
|  | Antibiotics (nr=3) | 2 | 66.67% | 1.09 | 0.10, 12.39 | 0.944 |
|  | Other disinfectant (nr=8) | 6 | 75.00% | 1.64 | 0.32, 8.47 | 0.557 |
|  | Does Not Treat Navels (nr=33) | 21 | 63.63% | 0.96 | 0.43, 2.13 | 0.910 |
|  |  |  |  |  |  |  |
|  | **Navel Treatment Application (nr=163)** | **106** | **65.03%** |  |  | **0.998** |
|  | Spray (baseline) (nr=69) | 45 | 65.22% |  |  |  |
|  | Dip (nr=58) | 38 | 65.52% | 1.01 | 0.49, 2.11 | 0.972 |
|  | Other (nr=3) | 2 | 66.67% | 1.07 | 0.09, 12.37 | 0.959 |
|  | Does Not Treat Navels (nr=33) | 21 | 63.63% | 0.93 | 0.39, 2.21 | 0.876 |
|  |  |  |  |  |  |  |
|  | **Age of Navel Treatment (nr=163)** | **106** | **65.03%** |  |  | **n/a** |
|  | Within 2 hours of birth (immediately) (nr=111) | 71 | 63.96% |  |  |  |
|  | Between 2 and 12 hours of birth (nr=17) | 12 | 70.59% |  |  |  |
|  | Other (nr=2) | 2 | 100.00% |  |  |  |
|  | Does Not Treat Navels (nr=33) | 21 | 63.63% |  |  |  |
|  |  |  |  |  |  |  |
|  | **Ear Tagging (nr=160)** | **104** | **65.00%** |  |  | **0.005** |
|  | No (baseline) (nr=87) | 65 | 74.71% |  |  |  |
|  | Yes (nr=73) | 39 | 53.42% | 2.58 | 1.32, 5.02 | 0.005 |
|  |  |  |  |  |  |  |
|  | **Cleaning Ear Tags (nr=164)** | **107** | **65.24%** |  |  | **0.004** |
|  | Yes (baseline) (nr=39) | 17 | 43.59% |  |  |  |
|  | No (nr=38) | 25 | 65.79% | 2.49 | 0.99, 6.26 | 0.053 |
|  | Does Not Ear Tag (nr=87) | 65 | 74.71% | 3.82 | 1.72, 8.48 | 0.001 |
|  |  |  |  |  |  |  |
|  | **Age of Ear Tagging (nr=73)** | **40** | **54.79%** | **1.00** | **0.99, 1.01** | **0.496** |
|  |  |  |  |  |  |  |
|  | **Castration (nr=161)** | **104** | **64.60%** |  |  | **0.315** |
|  | No (baseline) (nr=54) | 32 | 59.26% |  |  |  |
|  | Yes (nr=107) | 72 | 67.29% | 1.41 | 0.72, 2.78 | 0.315 |
|  |  |  |  |  |  |  |
|  | **Cleaning Castration Equipment (nr=162)** | **105** | **64.81%** |  |  | **0.451** |
|  | Yes (baseline) (nr=38) | 24 | 63.16% |  |  |  |
|  | No (nr=70) | 49 | 70.00% | 1.36 | 0.59, 3.13 | 0.469 |
|  | Does Not Castrate (nr=54) | 32 | 59.26% | 0.85 | 0.36, 1.99 | 0.706 |
|  |  |  |  |  |  |  |
|  | **Age of Castration (nr=162)** | **105** | **64.81%** |  |  | **0.275** |
|  | Within 24 Hours of Birth (baseline) (nr=40) | 30 | 75.00% |  |  |  |
|  | Over 24 hours (nr=68) | 43 | 63.24% | 0.57 | 0.24, 1.37 | 0.210 |
|  | Does Not Castrate (nr=54) | 32 | 59.26% | 0.49 | 0.20, 1.19 | 0.114 |
|  |  |  |  |  |  |  |
|  | **Tail Docking (nr=162)** | **106** | **65.43%** |  |  | **0.537** |
|  | No (baseline) (nr=36) | 22 | 61.11% |  |  |  |
|  | Yes (nr=126) | 84 | 66.67% | 1.27 | 0.59, 2.74 | 0.537 |
|  |  |  |  |  |  |  |
|  | **Cleaning Tail Docking Equipment (nr=164)** | **107** | **65.24%** |  |  | **0.790** |
|  | Yes (baseline) (nr=42) | 27 | 64.29% |  |  |  |
|  | No (nr=86) | 58 | 67.44% | 1.15 | 0.53, 2.50 | 0.723 |
|  | Does Not Dock (nr=36) | 22 | 61.11% | 0.87 | 0.35, 2.19 | 0.772 |
|  |  |  |  |  |  |  |
|  | **Age of Tail Docking (nr=164)** | **107** | **65.24%** |  |  | **0.333** |
|  | Within 24 Hours of Birth (baseline) (nr=44) | 31 | 70.45% |  |  |  |
|  | Between 1 day and 1 week old (nr=71) | 43 | 60.56% | 0.64 | 0.29, 1.44 | 0.283 |
|  | Older than 1 week (nr=13) | 11 | 84.62% | 2.31 | 0.45, 11.89 | 0.318 |
|  | Does Not Dock (nr=36) | 22 | 61.11% | 0.66 | 0.26, 1.67 | 0.380 |
