# Supplementary material for: A cross-sectional survey of farmer reported prevalence and farm management practices associated with neonatal infectious arthritis (“joint ill”) in lambs, on UK sheep farms
Source: Front Vet Sci. 2024 Dec 23;11:1489751. doi: 10.3389/fvets.2024.1489751 (PMC11701153; doi:10.3389/fvets.2024.1489751)
Supplement: Supplementary file 5 [file Table_5.DOCX]

**Supplementary Material 5: Full univariable results for the all flocks dataset.**

| Category | Variable  (nr= number of responses) | Number of farms with Joint Ill | Percentage of farms with Joint Ill | Odds Ratio | 95% CI | P value |
| --- | --- | --- | --- | --- | --- | --- |
| Ewe Factors | **Age of Ewes (nr=319)** | **203** | **63.64%** |  |  | **0.264** |
|  | Majority over 5 years old (baseline) (nr=6) | 3 | 50.00% |  |  |  |
|  | 1-2 years old (nr=46) | 2 | 4.35% | 0.50 | 0.05, 5.15 | 0.560 |
|  | Mixed ages (nr=307) | 198 | 64.50% | 1.82 | 0.36, 9.15 | 0.469 |
|  |  |  |  |  |  |  |
|  | **Body Condition Score Target (nr=170)** | **108** | **63.53%** |  |  | **0.428** |
|  | ≤3 (baseline) (nr=103) | 63 | 61.17% |  |  |  |
|  | >3 (nr=67) | 45 | 67.16% | 1.30 | 0.68, 2.48 | 0.428 |
|  |  |  |  |  |  |  |
|  | **‘Dagging’ Ewes (nr=319)** | **203** | **63.64%** |  |  | **0.308** |
|  | Yes (baseline) (nr=129) | 88 | 68.22% |  |  |  |
|  | No (nr=94) | 59 | 62.77% | 0.79 | 0.45, 1.37 | 0.397 |
|  | Only if dirty (nr=96) | 56 | 58.33% | 0.65 | 0.38, 1.13 | 0.128 |
|  |  |  |  |  |  |  |
|  | **Pure Bred vs. Cross Bred Ewes (nr=317)** | **202** | **63.72%** |  |  | **<0.001** |
|  | Cross bred (baseline) (nr=193) | 139 | 72.02% |  |  |  |
|  | Pure bred (nr=124) | 63 | 50.81% | 0.40 | 0.25, 0.64 | <0.001 |
|  |  |  |  |  |  |  |
| Farm Factors | **Flock Type (nr=305)** | **197** | **64.59%** |  |  | **0.057** |
|  | Lowland (baseline) (nr=194) | 116 | 59.79% |  |  |  |
|  | Upland (nr=100) | 74 | 74.00% | 1.91 | 1.13, 3.26 | 0.017 |
|  | Mountain (nr=11) | 7 | 63.64% | 1.18 | 0.33, 4.16 | 0.800 |
|  |  |  |  |  |  |  |
|  | **Organic vs. Non-organic (nr=322)** | **206** | **63.98%** |  |  | **0.900** |
|  | Non-Organic (baseline) (nr=306) | 196 | 64.05% |  |  |  |
|  | Organic (nr=16) | 10 | 62.50% | 0.94 | 0.33, 2.64 | 0.900 |
|  |  |  |  |  |  |  |
|  | **Lambing Duration (weeks) (nr=317)** | **202** | **63.72%** | **1.16** | **1.03, 1.30** | **0.013** |
|  |  |  |  |  |  |  |
|  | **Lambing Period (nr=316)** | **201** | **63.61%** |  |  | **0.016** |
|  | Middle (baseline) (Feb-Mar) (nr=173) | 116 | 67.05% |  |  |  |
|  | Early (Sep-Jan) (nr=23) | 8 | 34.78% | 0.26 | 0.11, 0.65 | 0.004 |
|  | Late (Apr-May) (nr=120) | 77 | 64.17% | 0.88 | 0.54, 1.44 | 0.609 |
|  |  |  |  |  |  |  |
|  | **Scanning % (nr=245)** | **151** | **61.63%** |  |  | **0.792** |
|  | 151-200 (baseline) (nr=185) | 116 | 62.70% |  |  |  |
|  | < or equal to 150 (nr=37) | 21 | 56.76% | 0.78 | 0.38, 1.60 | 0.498 |
|  | 201+ (nr=23) | 14 | 60.87% | 0.93 | 0.38, 2.25 | 0.864 |
|  |  |  |  |  |  |  |
|  | **No. Ewes Lambed (nr=307)** | **195** | **63.52%** |  |  | **<0.001** |
|  | 1-100 (baseline) (nr=80) | 30 | 37.50% |  |  |  |
|  | 101-300 (nr=74) | 43 | 58.11% | 2.31 | 1.21, 4.41 | 0.011 |
|  | 301-600 (nr=79) | 58 | 73.42% | 4.60 | 2.35, 9.03 | <0.001 |
|  | 601+ (nr=74) | 64 | 86.49% | 10.67 | 4.77, 23.87 | <0.001 |
|  |  |  |  |  |  |  |
|  | **Lambs Born Alive (nr=304)** | **190** | **62.50%** |  |  | **<0.001** |
|  | 1-160 (baseline) (nr=78) | 28 | 35.90% |  |  |  |
|  | 161-500 (nr=77) | 45 | 58.44% | 2.51 | 1.31, 4.80 | 0.005 |
|  | 501-1000 (nr=78) | 56 | 71.79% | 4.55 | 2.31, 8.94 | <0.001 |
|  | 1001+ (nr=71) | 61 | 85.92% | 10.89 | 4.83, 24.56 | <0.001 |
|  |  |  |  |  |  |  |
| Hygiene Factors | **Cleaning Stomach Tubes (nr=319)** | **203** | **63.64%** |  |  | **0.052** |
|  | Yes (baseline) (=226) | 153 | 67.70% |  |  |  |
|  | No (nr=6) | 4 | 66.67% | 0.95 | 0.17, 5.33 | 0.957 |
|  | Does Not Supplement (nr=87) | 46 | 52.87% | 0.54 | 0.32, 0.89 | 0.015 |
|  |  |  |  |  |  |  |
|  | **Freq. of Cleaning Stomach Tubes (nr=319)** | **203** | **63.64%** |  |  | **0.007** |
|  | Between each lamb and/or ewe (baseline) (nr=186) | 118 | 63.44% |  |  |  |
|  | Daily (nr=36) | 31 | 86.11% | 3.57 | 1.33, 9.62 | 0.012 |
|  | Does Not or Infrequently Cleans Stomach Tubes (nr=10) | 8 | 80.00% | 2.31 | 0.48, 11.17 | 0.300 |
|  | Does Not Supplement (nr=87) | 46 | 52.87% | 0.65 | 0.39, 1.08 | 0.098 |
|  |  |  |  |  |  |  |
|  | **Cleaning Bottles (nr=318)** | **202** | **63.52%** |  |  | **n/a** |
|  | Yes (baseline) (nr=228) | 153 | 67.11% |  |  |  |
|  | No (nr=3) | 3 | 100.00% |  |  |  |
|  | Does Not Supplement (nr=87) | 46 | 52.87% |  |  |  |
|  |  |  |  |  |  |  |
|  | **Freq. of Cleaning Bottles (nr=317)** | **201** | **63.41%** |  |  | **0.014** |
|  | Between each lamb (baseline) (nr=144) | 89 | 61.81% |  |  |  |
|  | Daily (nr=79) | 61 | 77.22% | 2.09 | 1.12, 3.91 | 0.020 |
|  | Does Not or Infrequently Cleans Bottles (nr=7) | 5 | 71.43% | 1.54 | 0.29, 8.24 | 0.611 |
|  | Does Not Supplement (nr=87) | 46 | 52.87% | 0.69 | 0.41, 1.19 | 0.183 |
|  |  |  |  |  |  |  |
|  | **Freq. of Cleaning Lambing Ropes and Head Snares (nr=305)** | **193** | **63.28%** |  |  | **0.671** |
|  | Between each use (baseline) (nr=257) | 160 | 62.26% |  |  |  |
|  | Daily (nr=27) | 19 | 70.37% | 1.44 | 0.61, 3.42 | 0.408 |
|  | Weekly (nr=21) | 14 | 66.67% | 1.21 | 0.47, 3.11 | 0.688 |
|  |  |  |  |  |  |  |
|  | **Wearing Gloves (nr=321)** | **205** | **63.86%** |  |  | **0.392** |
|  | Yes (baseline) (nr=129) | 86 | 66.67% |  |  |  |
|  | No (nr=192) | 119 | 61.98% | 0.82 | 0.51, 1.30 | 0.392 |
|  |  |  |  |  |  |  |
|  |  |  |  |  |  |  |
|  | **Washing Hands (nr=321)** | **205** | **63.86%** |  |  | **0.033** |
|  | Yes (baseline) (nr=235) | 140 | 59.57% |  |  |  |
|  | No (nr=21) | 16 | 76.19% | 2.17 | 0.77, 6.13 | 0.143 |
|  | Sometimes (nr=65) | 49 | 75.38% | 2.08 | 1.12, 3.87 | 0.021 |
|  |  |  |  |  |  |  |
|  | **Method of Hand Washing (nr=312)** | **199** | **63.78%** |  |  | **0.764** |
|  | Disinfectant (baseline) (nr=45) | 27 | 60.00% |  |  |  |
|  | Soap and Water (nr=172) | 106 | 61.63% | 1.07 | 0.55, 2.09 | 0.842 |
|  | Alcohol gel (nr=6) | 4 | 66.67% | 1.33 | 0.22, 8.06 | 0.754 |
|  | Just water (nr=11) | 8 | 72.73% | 1.78 | 0.42, 7.62 | 0.438 |
|  | Combination of methods (nr=57) | 38 | 66.67% | 1.33 | 0.59, 3.00 | 0.487 |
|  | Does Not Wash Hands (nr=21) | 16 | 76.19% | 2.13 | 0.66, 6.86 | 0.204 |
|  |  |  |  |  |  |  |
| Lamb Factors | **Using Preventative Measures for JI (nr=321)** | **206** | **64.17%** |  |  | **0.009** |
|  | Yes (baseline) (nr=214) | 148 | 69.16% |  |  |  |
|  | No (nr=107) | 58 | 54.21% | 0.53 | 0.33, 0.85 | 0.009 |
|  |  |  |  |  |  |  |
|  | **Antibiotics as Preventative Measures (nr=320)** | **205** | 64.06% |  |  | **0.014** |
|  | Yes (baseline) (nr=34) | 20 | 58.82% |  |  |  |
|  | No (nr=179) | 127 | 70.95% | 1.71 | 0.80, 3.64 | 0.164 |
|  | Does Not Use Preventative Measures (nr=107) | 58 | 54.21% | 0.83 | 0.38, 1.81 | 0.637 |
|  |  |  |  |  |  |  |
|  | **Lambs Given Antibiotics for Prevention (nr=319)** | **205** | **64.26%** |  |  | **0.426** |
|  | Does Not Use Antibiotics/Preventative Measures (baseline) (nr=266) | 170 | 82.93% |  |  |  |
|  | Metaphylactic Treatment (nr=8) | 7 | 87.50% | 3.95 | 0.48, 32.61 | 0.202 |
|  |  |  |  |  |  |  |
|  | Prophylactic Treatment (nr=45) | 28 | 62.22%% | 0.93 | 0.48, 1.79 | 0.828 |
|  | Does Not Use Preventative Measures (nr=106) | 58 | 54.72% | 0.17 | 0.02, 1.45 | 0.106 |
|  |  |  |  |  |  |  |
|  | **Monitoring Colostrum (nr=319)** | **203** | **63.64%** |  |  | **0.806** |
|  | Yes (baseline) (nr=272) | 175 | 64.34% |  |  |  |
|  | No (nr=19) | 11 | 57.89% | 0.76 | 0.30, 1.96 | 0.573 |
|  | Sometimes (nr=28) | 17 | 60.71% | 0.86 | 0.39, 1.90 | 0.704 |
|  |  |  |  |  |  |  |
|  | **Supplementing Colostrum (nr=321)** | **205** | **63.86%** |  |  | **0.013** |
|  | Yes (baseline) (nr=234) | 159 | 67.95% |  |  |  |
|  | No (nr=87) | 46 | 52.87% | 0.53 | 0.32, 0.88 | 0.013 |
|  |  |  |  |  |  |  |
|  | **Treating Navels (nr=318)** | **203** | **63.84%** |  |  | **0.718** |
|  | Yes (baseline) (nr=282) | 181 | 64.18% |  |  |  |
|  | No (nr=36) | 22 | 61.11% | 0.88 | 0.43, 1.79 | 0.718 |
|  |  |  |  |  |  |  |
|  | **Freq. of Navel Treatment (nr=312)** | **201** | **64.42%** |  |  | **0.669** |
|  | Once (baseline) (nr=191) | 121 | 63.35% |  |  |  |
|  | Twice (nr=85) | 58 | 68.24% | 1.24 | 0.72, 2.14 | 0.433 |
|  | Does Not Treat Navels (nr=36) | 22 | 61.11% | 0.91 | 0.44, 1.89 | 0.799 |
|  |  |  |  |  |  |  |
|  | **Navel Treatment Used (nr=320)** | **204** | **63.75%** |  |  | **0.981** |
|  | Iodine (baseline) (nr=260) | 167 | 64.23% |  |  |  |
|  | Antibiotics (nr=3) | 2 | 66.67% | 1.11 | 0.10, 12.45 | 0.930 |
|  | Other disinfectant (nr=21) | 13 | 61.90% | 0.91 | 0.36, 2.26 | 0.831 |
|  | Does Not Treat Navels (nr=36) | 22 | 61.11% | 0.88 | 0.43, 1.79 | 0.715 |
|  |  |  |  |  |  |  |
|  | **Navel Treatment Application Method (nr=320)** | **204** | **63.75%** |  |  | **0.950** |
|  | Spray (baseline) (nr=158) | 103 | 65.19% |  |  |  |
|  | Dip (nr=120) | 75 | 62.50% | 0.89 | 0.54, 1.46 | 0.644 |
|  | Other (nr=6) | 4 | 66.67% | 1.07 | 0.19, 6.02 | 0.941 |
|  | Does Not Treat Navels (nr=36) | 22 | 61.11% | 0.84 | 0.40, 1.75 | 0.645 |
|  |  |  |  |  |  |  |
|  | **Age of Navel Treatment (nr=320)** | **204** | **63.75%** |  |  | **0.925** |
|  | Within 2 hours of birth (immediately) (nr=255) | 162 | 63.53% |  |  |  |
|  | Between 2 and 12 hours of birth (nr=23) | 16 | 69.57% | 1.31 | 0.52, 3.31 | 0.564 |
|  | Other (nr=6) | 4 | 66.67% | 1.15 | 0.21, 6.39 | 0.875 |
|  | Does Not Treat Navels (nr=36) | 22 | 61.11% | 0.90 | 0.44, 1.85 | 0.778 |
|  |  |  |  |  |  |  |
|  | **Ear Tagging (nr=316)** | **201** | **63.61%** |  |  | **0.020** |
|  | No (baseline) (nr=173) | 120 | 69.36% |  |  |  |
|  | Yes (nr=143) | 81 | 56.64% | 0.58 | 0.36, 0.92 | 0.020 |
|  |  |  |  |  |  |  |
|  | **Cleaning Ear Tags (nr=321)** | **205** | **63.86%** |  |  | **0.026** |
|  | Yes (baseline) (nr=76) | 39 | 51.32% |  |  |  |
|  | No (nr=72) | 46 | 63.89% | 0.60 | 0.31, 1.15 | 0.123 |
|  | Does Not Ear Tag (nr=173) | 120 | 69.36% | 1.28 | 0.72, 2.28 | 0.404 |
|  |  |  |  |  |  |  |
|  | **Age of Ear Tagging (nr=140)** | **80** | **57.14%** | **1.00** | **0.99, 1.01** | **0.801** |
|  |  |  |  |  |  |  |
|  | **Castration (nr=316)** | **200** | **63.29%** |  |  | **0.245** |
|  | No (baseline) (nr=107) | 63 | 58.88% |  |  |  |
|  | Yes (nr=209) | 137 | 65.55% | 1.33 | 0.82, 2.15 | 0.245 |
|  |  |  |  |  |  |  |
|  | **Cleaning Castration Equipment (nr=319)** | **203** | **63.64%** |  |  | **0.312** |
|  | Yes (baseline) (nr=71) | 44 | 61.97% |  |  |  |
|  | No (nr=141) | 96 | 68.09% | 1.31 | 0.72, 2.38 | 0.376 |
|  | Does Not Castrate (nr=107) | 63 | 58.88% | 0.88 | 0.48, 1.63 | 0.680 |
|  |  |  |  |  |  |  |
|  | **Age of Castration (nr=319)** | **203** | **63.64%** |  |  | **0.013** |
|  | Within 24 hours of birth (baseline) (nr=78) | 59 | 75.64% |  |  |  |
|  | Between 1 day and 1 week old (nr=122) | 70 | 57.38% | 0.43 | 0.23, 0.81 | 0.009 |
|  | Older than 1 week (nr=12) | 11 | 91.67% | 3.54 | 0.43, 29.24 | 0.240 |
|  | Does Not Castrate (nr=107) | 63 | 58.88% | 0.46 | 0.24, 0.88 | 0.019 |
|  |  |  |  |  |  |  |
|  | **Tail Docking (nr=317)** | **202** | **63.72%** |  |  | **0.036** |
|  | No (baseline) (nr=53) | 27 | 50.94% |  |  |  |
|  | Yes (nr=264) | 175 | 66.29% | 1.89 | 1.04, 3.44 | 0.036 |
|  |  |  |  |  |  |  |
|  | **Cleaning Tail Docking Equipment (nr=321)** | **205** | **63.86%** |  |  | **0.097** |
|  | Yes (baseline) (nr=79) | 51 | 64.56% |  |  |  |
|  | No (nr=189) | 127 | 67.20% | 1.13 | 0.65, 1.95 | 0.677 |
|  | Does Not Tail Dock (nr=53) | 27 | 50.94% | 0.57 | 0.28, 1.16 | 0.120 |
|  |  |  |  |  |  |  |
|  | **Age of Tail Docking (nr=320)** | **204** | **63.75%** |  |  | **0.008** |
|  | Between 1 day and 1 week old (baseline) (nr=153) | 91 | 59.48% |  |  |  |
|  | Within 24 hours of birth (nr=99) | 73 | 73.74% | 1.91 | 1.10, 3.32 | 0.021 |
|  | Older than 1 week (nr=15) | 13 | 86.67% | 4.43 | 0.97, 20.32 | 0.056 |
|  | Does Not Tail Dock (nr=53) | 27 | 50.94% | 0.71 | 0.38, 1.33 | 0.280 |
