# Supplementary material for: A cross-sectional survey of farmer reported prevalence and farm management practices associated with neonatal infectious arthritis (“joint ill”) in lambs, on UK sheep farms
Source: Front Vet Sci. 2024 Dec 23;11:1489751. doi: 10.3389/fvets.2024.1489751 (PMC11701153; doi:10.3389/fvets.2024.1489751)
Supplement: Supplementary file 6 [file Table_6.docx]

**Supplementary Material 6**

1. **Flock Attributes**

| Attribute |  | Percentage of Farms (number of farms) | Median |
| --- | --- | --- | --- |
| Flock type (N=316) | Lowland | 61% (193/316) |  |
|  | Upland | 35% (110/316) |  |
|  | Mountain/Hill | 4% (13/316) |  |
| Organic vs. non-organic  (N=316) | Organic | 5% (16/316) |  |
|  | Non-organic | 95% (300/316) |  |
| Flock size (N=318) |  |  | 320 (IQR, 100-650) |
| Cross-bred vs. Pure-bred (N=319) | Cross-bred | 61% (193/317) |  |
|  | Pure-bred | 39% (124/319) |  |
| Breed (N=317) | Texel | 9% (29/317) |  |
|  | Lleyn | 7% (22/317) |  |
|  | Mule | 6% (19/317) |  |
|  | North of England Mule | 4% (13/317) |  |
|  | Suffolk | 4% (13/317) |  |
|  | Other | 70% (221/317) |  |
| Age of ewes (N=319) | 1-2 years old | 2% (6/319) |  |
|  | >5 years old | 2% (6/319) |  |
|  | Mixed | 96% (307/319) |  |
| Lambing system (N=319) | Indoor | 51% (162/319) |  |
|  | Outdoor | 25% (80/319) |  |
|  | Mixed | 24% (77/319) |  |
| Months of lambing | January | 4% (21/319) |  |
|  | February | 13% (63/319) |  |
|  | March | 35% (112/319) |  |
|  | April | 39% (124/319) |  |
|  | May | 7% (34/319) |  |
|  | June | <1% (1/319) |  |
|  | September | <1% (1/319) |  |
|  | November | <1% (1/319) |  |
|  | December | 1% (6/319) |  |
| Scanning percentage (N=245) |  |  | 178% (IQR, 160-190) |
| Ewes lambed (N=307) |  |  | 300 (IQR, 95-600) |
| Lambs born alive (N=304) |  |  | 500 (IQR, 154-980) |

1. **NIA Case Characteristics**

| Characteristic |  | Percentage of Farms (number of responses) | Median |
| --- | --- | --- | --- |
| Farmer reported NIA incidence in 2020 (N=322) |  | 64% (206/322) |  |
| Median cases per farm (N=304) |  |  | 8.5 (IQR, 3-20) |
| Lambs born going on to develop NIA (N=304) |  |  | 1.4% (IQR, 0.8-2.6) |
| Systems reporting NIA (N=204) | Indoor | 63% (128/204) |  |
|  | Outdoor | 19% (39/204) |  |
|  | Mixed | 18% (37/204) |  |
| Time of NIA development (N=204) | Earlier into lambing | 12% (24/204) |  |
|  | Middle of lambing | 25% (51/204) |  |
|  | Later into lambing | 36% (73/204) |  |
|  | No specific time | 28% (56/204) |  |
| Age of NIA lambs (N=205) | 0-3 days old | 6% (12/205) |  |
|  | 4-7 days old | 26% (54/205) |  |
|  | 8-14 days old | 38% (78/205) |  |
|  | 15-28 days old | 24% (49/205) |  |
|  | >1 month old | 6% (12/205) |  |
| Lambs developing NIA (N=205) | Singles | 4% (8/205) |  |
|  | Twins | 24% (47/205) |  |
|  | Triplets | 16% (33/205) |  |
|  | Orphans | 9% (19/205) |  |
|  | Other | <1% (1/205) |  |

1. **Lambing Management Practices**

| System | Practice |  | Percentage of Farms (number of responses) | Median |
| --- | --- | --- | --- | --- |
| Outdoor | Lambing (N=322) | Outdoor only | 26% (84/322) |  |
|  |  | Mixed | 26% (84/322) |  |
|  |  | Total | 52% (168/322) |  |
|  | Providing shelter (N=162) | Yes | 27% (44/162) |  |
|  |  | No | 73% (118/162) |  |
|  | ‘Moving on’ of ewes and lambs (N=159) | Within 24 hours | 19% (31/159) |  |
|  |  | 1-3 days | 27% (43/159) |  |
|  |  | 4-7 days | 11% (17/159) |  |
|  |  | >1 week | 7% (11/159) |  |
|  |  | Set stock | 16% (25/159) |  |
|  |  | Varied | 20% (32/159) |  |
| Indoor | Lambing (N=309) | Indoor only | 47% (147/309) |  |
|  |  | Mixed | 25% (76/309) |  |
|  |  | Total | 72% (223/309) |  |
|  | Time housed (N=205) |  |  | 4 weeks (IQR, 2-6 weeks) |
|  | Group pen hygiene (N=231) | Yes | 72% (167/231) |  |
|  |  | No | 28% (64/231) |  |
|  | Use of mothering pens (N=234) | Yes | 94% (221/234) |  |
|  |  | No | <1% (1/234) |  |
|  |  | Sometimes | 5% (12/234) |  |
|  | Quantity of mothering pens (N=211) |  |  | 40 (IQR, 20-70) |
|  | Bedding in mothering pens (N=233) | Straw | 94% (218/233) |  |
|  |  | Shavings | 5% (12/233) |  |
|  |  | Other | 1% (3/233) |  |
|  | Mothering pen hygiene (N=325) | Top up bedding between ewes | 38% (123/325) |  |
|  |  | Cleaned between ewes | 34% (109/325) |  |
|  |  | Cleaned when visibly dirty or soiled | 13% (42/325) |  |
|  |  | Cleaned once per week | 1% (3/325) |  |
|  |  | Cleaned at end of season | 12% (38/325) |  |
|  |  | Other | 3% (8/325) |  |
|  | Disinfection of mothering pens (N=231) | Yes | 70% (161/231) |  |
|  |  | No | 30% (70/231) |  |
|  | Disinfectants used in mothering pens (N=167) | Lime | 43% (72/167) |  |
|  |  | Virkon | 12% (20/167) |  |
|  |  | Stalosan | 7% (12/167) |  |
|  |  | Sorgene | 6% (10/167) |  |
|  |  | FAM | 4% (7/167) |  |
|  | Time in mothering pens (N=232) | <24 hours | 27% (62/232) |  |
|  |  | 1-3 days | 35% (82/232) |  |
|  |  | 4-7 days | 2% (5/232) |  |
|  |  | Varied | 36% (83/232) |  |
|  | Use of nursery pens (N=233) | Yes | 56% (130/233) |  |
|  |  | No | 15% (36/233) |  |
|  |  | Sometimes | 29% (67/233) |  |
|  | Bedding in nursery pens (N=197) | Straw | 99% (195/197) |  |
|  |  | Shavings | 1% (2/197) |  |
|  | Nursery pen hygiene (N=283) | Top up bedding between ewes | 43% (123/283) |  |
|  |  | Cleaned between ewes | 6% (18/283) |  |
|  |  | Cleaned when visibly dirty or soiled | 14% (39/283) |  |
|  |  | Cleaned every day | 3% (8/283) |  |
|  |  | Cleaned every couple of days | 2% (7/283) |  |
|  |  | Cleaned once per week | 2% (5/283) |  |
|  |  | Cleaned at end of season | 26% (74/283) |  |
|  |  | Other | 3% (6/197) |  |
|  | Disinfection of nursery pens (N=195) | Yes | 39% (77/195) |  |
|  |  | No | 61% (11/195) |  |
|  | Disinfectants used in nursery pens (N=77) | Lime | 53% (41/77) |  |
|  |  | Virkon | 17% (13/77) |  |
|  | Time in nursery pens (N=198) | <24 hours | 13% (25/198) |  |
|  |  | 1-3 days | 44% (88/198) |  |
|  |  | 4-7 days | 9% (18/198) |  |
|  |  | >7 days | 6% (11/198) |  |
|  |  | Varied | 28% (56/198) |  |
|  | Total time indoors (N=233) | <24 hours | 10% (23/233) |  |
|  |  | 1-3 days | 45%, 104/233 |  |
|  |  | 4-7 days | 17% (39/233) |  |
|  |  | >7 days | 10% (24/233) |  |
|  |  | Varied | 18% (43/233) |  |
|  |  |  |  |  |
| All Flocks | Body Condition Scoring of Ewes (N=319) | Yes | 55% (176/319) |  |
|  |  | No | 45% (143/319) |  |
|  |  |  |  |  |
|  | Median Body Condition Score Target of Ewes (N=170) |  |  | 3 (IQR, 3-4) |
|  |  |  |  |  |
|  | Monitoring Ewes and Lambs for Colostrum Intake (N=319) | Yes | 85% (272/319) |  |
|  |  | No | 6% (19/319) |  |
|  |  | Sometimes | 9% (28/319) |  |
|  |  |  |  |  |
|  | Method of Colostrum Monitoring in Ewes and Lambs (N=327) | Checking lambs for fullness of stomach | 48% (156/327) |  |
|  |  | Checking for presence of colostrum in ewe udder | 25% (81/327) |  |
|  |  | Observing lambs suckling the ewe | 24% (80/327) |  |
|  |  | Blood test monitoring via a vet | 1% (2/327) |  |
|  |  | Other | 2% (8/327) |  |
|  |  |  |  |  |
|  | Farmers Supplementing Colostrum Intake of Lambs (N=321) | Yes | 73% (234/321) |  |
|  |  | No | (87/321) |  |
|  |  |  |  |  |
|  | Lambs given supplementary colostrum (N=540) | Lambs who do not suckle within 6 hours of birth | 26% (141/540) |  |
|  |  | Lambs who do not suckle within 24 hours | 4% (19/540) |  |
|  |  | Those born to old/poor body condition ewes | 23% (126/540) |  |
|  |  | Lambs who don’t immediately suckle | 15% (81/540) |  |
|  |  | Twins/doubles | 1% (4/540) |  |
|  |  | Triples | 25% (136/540) |  |
|  |  | All lambs | 2% (9/540) |  |
|  |  | Other | 4% (24/540) |  |
|  |  |  |  |  |
|  | Source of Supplementary Colostrum (N=515) | Commercial colostrum | 40% (208/515) |  |
|  |  | Ewe’s own colostrum | 26% (132/515) |  |
|  |  | Fresh colostrum from another ewe | 20% (104/515) |  |
|  |  | Cow colostrum | 6% (29/515) |  |
|  |  | Frozen colostrum from another ewe | 8% (41/515) |  |
|  |  | Other | <1% (1/515) |  |
|  |  |  |  |  |
|  | Amount of Supplementary Colostrum given to Lambs (N=217) |  |  | 120ml (IQR, 75-150ml) |
|  |  |  |  |  |
|  | Cleaning of Stomach Tubes (N=232) | Yes | (226/232) |  |
|  |  | No | (6/232) |  |
|  |  |  |  |  |
|  | Frequency of Cleaning Stomach Tubes (N=238) | Between each lamb | 64% (152/238) |  |
|  |  | Between each ewe | 15% (35/238) |  |
|  |  | Daily | 19% (45/238) |  |
|  |  | Weekly | 2% (4/238) |  |
|  |  | After dosing sick lambs | 1% (2/238) |  |
|  |  |  |  |  |
|  | Cleaning of Bottles and Teats (N=231) | Yes | 99% (28/231) |  |
|  |  | No | (3/228) |  |
|  |  |  |  |  |
|  | Frequency of Cleaning Bottles and Teats (N=246) | Between each lamb | 59% (144/246) |  |
|  |  | Daily | 39% (95/246) |  |
|  |  | Weekly | 2% (4/246) |  |
|  |  | After feeding sick lambs | 1% (3/246) |  |
|  |  |  |  |  |
|  | No. of Staff Members involved in Lambing (N=319) |  |  | 2 (IQR, 2-3) |
|  |  |  |  |  |
|  | Wearing Gloves whilst Lambing (N=321) | Yes | 40% (129/321) |  |
|  |  | No | 28% (90/312) |  |
|  |  | Sometimes | 32% (102/321) |  |
|  |  |  |  |  |
|  | Staff Hand Washing during Lambing (N=321) | Yes | 73% (235/321) |  |
|  |  | No | 7% (21/321) |  |
|  |  | Sometimes | 20% (65/321) |  |
|  |  |  |  |  |
|  | Method of Hand Washing (N=370) | Soap and Water | 61% (226/370) |  |
|  |  | Disinfectant | 24% (88/37) |  |
|  |  | Alcohol gel | 8% (28/370) |  |
|  |  | Water | 7% (26/370) |  |
|  |  | Other | <1% (2/370) |  |
|  |  |  |  |  |
|  | ‘Dagging’* of Ewes (N=319) | Ewes routinely dagged | 40% (129/319) |  |
|  |  | Ewes dagged if required | 30% (96/319) |  |
|  |  | Ewes not dagged | 30% (94/319)_ |  |
|  |  |  |  |  |
|  | Cleaning of Head Ropes (N=305) | Cleaned between each use | 84% (257/305) |  |
|  |  | Cleaned daily | 9% (27/305) |  |
|  |  | Cleaned weekly | 7% (21/305) |  |
|  |  |  |  |  |
|  | Treating Lambs Navels (N=318) | Yes | 89% (282/318) |  |
|  |  | No | 11% (36/318) |  |
|  |  |  |  |  |
|  | Frequency of Treating Lambs Navels (N=276) | Once | 69% (191/276) |  |
|  |  | Twice | 31% (85/276) |  |
|  |  |  |  |  |
|  | Timing of Lamb Navel Treatment (N=322) | Within 2 hours of birth | 79% (255/322) |  |
|  |  | Within 6 hours of birth | 14% (44/322) |  |
|  |  | Within 12 hours of birth | 5% (15/322) |  |
|  |  | Other | 2% (8/322) |  |
|  |  |  |  |  |
|  | Method of Navel Treatment (N=284) | Spray | (158/284) |  |
|  |  | Dip | (120/284) |  |
|  |  | Other | (6/284) |  |
|  |  |  |  |  |
|  |  |  |  |  |
|  | Treatment Used for Lambs Navels (N=284) | Iodine | 92% (260/284) |  |
|  |  | Topical antibiotics | 1% (3/284) |  |
|  |  | Other disinfectant | 7% (21/284) |  |
|  |  |  |  |  |
|  | Ear Tagging of Lambs (N=316) | Yes | (143/316) |  |
|  |  | No | 55% (173/316) |  |
|  |  |  |  |  |
|  | Age of Ear Tagging Lambs (N=140) |  |  | 2 days old (IQR, 1-7 days old) |
|  |  |  |  |  |
|  | Cleaning of Ear Tagging Equipment (N=148) | Yes | 51% (76/148) |  |
|  |  | No | (72/148) |  |
|  |  |  |  |  |
|  | Castration of Lambs (N=316) | Yes | 66% (209/316) |  |
|  |  | No | 34% (107/316) |  |
|  |  |  |  |  |
|  | Age of Castrating Lambs (N=212) | Within 6 hours of birth | 3% (7/212) |  |
|  |  | Within 24 hours of birth | 33% (71/212) |  |
|  |  | Between one day and one week of birth | 58% (122/212) |  |
|  |  | Over a week since birth | 6% (12/212) |  |
|  |  |  |  |  |
|  | Cleaning of Castration Equipment (N=212) | Yes | 33% (71/212) |  |
|  |  | No | 67% (141/212) |  |
|  |  |  |  |  |
|  | Tail Docking of Lambs (N=317) | Yes | 83% (264/317) |  |
|  |  | No | 17% (53/317) |  |
|  |  |  |  |  |
|  | Age of Tail Docking Lambs (N=267) | Within 6 hours of birth | 4% (11/267) |  |
|  |  | Within 24 hours of birth | 33% (88/267) |  |
|  |  | Between one day and one week of birth | 57% (153/267) |  |
|  |  | Over a week since birth | 6% (15/267) |  |
|  |  |  |  |  |
|  | Cleaning of Tail Docking Equipment (N=268) | Yes | 29% (79/268) |  |
|  |  | No | 71% (189/268) |  |

* ‘Dagging’ refers to the removal of dry faeces from wool on rear of sheep.
